# Research on the EEMD-SE-IWTD Combined Noise Reduction Method for High-Speed Transient Complex Features in Acceleration Signals

**DOI:** 10.3390/s25195940

**Published:** 2025-09-23

**Authors:** Huifa Shi, Shaojie Ma, Feiyin Li, Tong Tang, Kunming Jia, He Zhang

**Affiliations:** School of Mechanical Engineering, Nanjing University of Science and Technology, Nanjing 210094, China; shihf@njust.edu.cn (H.S.); tang_t@njust.edu.cn (T.T.); kunming_jia@163.com (K.J.); hezhangz@njust.edu.cn (H.Z.)

**Keywords:** high-speed transient complex features, acceleration, combined noise reduction, ensemble empirical mode decomposition (EEMD), sample entropy (SE), improved wavelet threshold denoising (IWTD)

## Abstract

Traditional noise reduction methods often struggle to balance noise suppression with the preservation of transient features in acceleration signals, especially when dealing with high-speed transient data. This study proposes a novel noise reduction method combining ensemble empirical mode decomposition (EEMD), sample entropy (SE), and improved wavelet threshold denoising (IWTD) to address the issue. The method utilizes EEMD to decompose the signal into intrinsic mode functions (IMFs) and a residual term. By setting an SE threshold (SE = 0.3), it effectively differentiates noise-dominated components from those containing significant transient features. IWTD is then applied to the noise-dominated components, and the processed components are reconstructed to yield the denoised signal. A baseline signal is generated in the lab, and noise is added to create the test set. The results show that this method achieves optimal noise reduction performance. Its effectiveness is validated through the output signal-to-noise ratio, root mean square error, and correlation coefficient. Overall, this method enhances noise reduction performance while preserving transient features. The method has been validated using real multi-layer penetration acceleration signals, supporting subsequent penetration layer identification and inversion analysis of the penetration process.

## 1. Introduction

High-speed transient complex features generally pertain to rapid and unpredictable transient phenomena observed in signals. The collision and penetration of a high-speed moving object with an impacted object are dynamic processes characterized by their swift and transient nature [1]. Specifically, acceleration signals with high-speed transient features can reflect the impact and penetration process, while the complex features manifest as nonlinear, non-stationary noise. For example, during a missile’s penetration of a multi-story building, the acceleration signals exhibit high-speed transient complex features [2]. In the time domain, these features appear as the superposition of the effective transient components of the impact and multiple oscillatory interference signals. In the frequency domain, they manifest as broadband noise that masks the main frequency band. This phenomenon can be attributed to electromagnetic interference from the external environment and noise caused by internal circuit oscillation of electronic components. Additionally, complex dynamic oscillatory interference from the mechanical structure of the moving object contributes to the noise [3,4]. These complex noises significantly obscure the characteristic signals of high-speed transients, particularly those of acceleration signals at the millisecond level. As a result, accurately identifying the high-speed transient features in the acceleration signals of high-speed moving objects passing through target objects becomes extremely difficult. These transient characteristics (such as the pulse width, amplitude, and interval) serve as the key basis for identifying the number of target object layers during the penetration process and for reconstructing the penetration process. Therefore, effective noise reduction of high-speed transient complex feature acceleration signals, while preserving key features, is crucial for signal feature identification and analysis.

The noise in acceleration signals with complex high-speed transients is primarily concentrated in the high-frequency range. Traditional noise reduction methods include low-pass filtering, wavelet threshold filtering, singular value decomposition, and empirical mode decomposition. Low-pass filtering effectively removes high-frequency noise. For instance, Forrestal et al. and Sandia National Laboratories used a 1 kHz low-pass filter to process acceleration signals [5,6]. Warren et al. analyzed acceleration signals in the 0–1.5 kHz frequency range [4], while the U.S. Army Corps of Engineers Waterways Experiment Station used a 4 kHz filter to remove noise [7]. Liu et al. analyzed acceleration signals in the 0–5 kHz frequency range [8]. However, this method depends on filter frequency selection and cannot achieve fully adaptive signal processing [9]. Since its introduction by Donoho et al. in 1995 [10], wavelet threshold filtering has been widely applied due to its adaptability and high resolution in time-varying signal processing. This method reduces noise in compression coefficients using global thresholds, effectively suppressing interference noise in signals. However, under conditions of high-intensity complex noise, the reconstructed signal may exhibit oscillations, leading to signal deviation [11,12]. Singular value decomposition (SVD) separates useful information from noise-contaminated signals by extracting the effective information and energy distribution of the signal. Experimental results show that SVD outperforms wavelet threshold filtering in noise reduction. The effectiveness of this method depends on the reasonable selection of the decomposition order and the number of rows in the submatrix, although no unified standard has yet been established [13,14].

Empirical mode decomposition (EMD) was introduced by Huang et al. in 1998 [15] as an effective method for processing non-stationary signals. However, EMD is prone to mode aliasing during signal decomposition, which results in less accurate decomposition. To address this issue, ensemble empirical mode decomposition (EEMD) was proposed in 2009 [16], which suppresses mode aliasing by adding white noise, thereby significantly improving the accuracy of signal decomposition. Building on EEMD, Zhang et al. [17] proposed an enhanced denoising algorithm that designs different low-pass filters based on intrinsic mode functions (IMFs) and combines them with enhanced denoising techniques to process sensor measurement data. Experimental results demonstrate that this method can effectively remove noise and eliminate the impact of installation structures on sensor performance, significantly improving sensor performance in practice. With the continuous advancement of EEMD technology, several improved methods have been proposed, including CEEMD [18], CEEMDAN [19], impCEEMDAN [20], and ECEEMDAN [21]. These methods show significant advantages in noise removal performance and have been widely applied in the noise reduction processing of complex signals. In recent years, fusion denoising methods based on EEMD have also made significant progress, such as EEMD-SVD [22], EEMD-MSPCA [23], EEMD-SSA [24], and EEMD-VMD-IMWOA [25]. However, despite the good results achieved by these optimization methods in noise reduction, the partitioning of IMF components in EEMD and the complete removal of noise IMF components remain key challenges. Therefore, maintaining the integrity of signal features during noise reduction continues to be a significant challenge in current research.

In response to the challenge of identifying complex features in high-speed transient multi-layer acceleration signals and the potential loss of effective features when noise components are removed using existing noise reduction methods, this paper proposes an EEMD-SE-IWTD combined noise reduction method. The method consists of three main steps: signal decomposition, mode division, and noise processing. During signal decomposition, EEMD is used to generate IMF components, effectively avoiding the interference of end-point effects on the results. In the mode division step, a separation algorithm based on sample entropy thresholds (SE = 0.3) is employed to distinguish noise-dominated IMF components (SE > 0.3) from IMF components dominated by effective transient features (SE ≤ 0.3). For noise processing, IWTD is used to perform precise noise reduction in the complex noise-dominated IMF components. Finally, the denoised signal is reconstructed. This method enhances noise reduction performance while successfully preserving the key features of the signal, making it highly applicable for denoising high-speed transient complex features in multi-layer acceleration signals. Additionally, this method may also be implemented in domains characterized by substantial noise signals, such as mechanical fault diagnosis and geological exploration. It is instrumental in developing more reliable sensor systems for complex dynamic environments and holds significant practical value.

## 2. Features and Noise Reduction Limitations in Acceleration Signals

### 2.1. Multi-Layer Penetration Acceleration Signal Feature

Multi-layer penetration acceleration signals are generated through the interaction between a high-speed moving object and the impacted target. These signals are acquired utilizing devices such as pressure sensors and accelerometers. The fundamental characteristics of these signals can be described in terms of the time domain and frequency domain.

**Figure 1:**
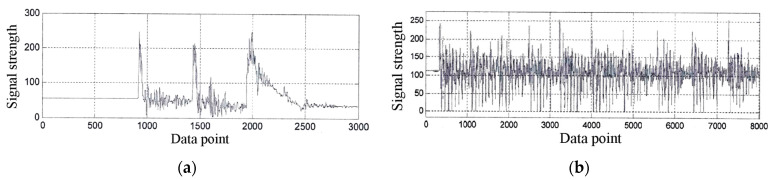
Time-domain characteristics of multi-layer penetration acceleration signals [26]. (**a**) The measured acceleration curve of a 125 mm caliber shell penetrating three-layer targets (clear multi-layer penetration acceleration signal). (**b**) The measured acceleration curve of a ground-penetrating rocket penetrating a nine-layer target (high-speed transient complex feature multi-layer penetration acceleration signal).

Multi-layer penetration acceleration signals capture time-domain characteristics, manifested as sudden changes or variations in amplitude, reflecting the occurrence of the penetration process. In actual signals, these changes are often closely related to the magnitude of the interaction force between the two objects. Multi-layer penetration acceleration signals primarily take two forms. One is a multi-layer penetration acceleration signal with a relatively clear envelope. At the same time, the other is a high-speed transient complex feature multi-layer penetration acceleration signal with oscillatory responses that are closely coupled. Figure 1 shows the time-domain characteristics of the two types of multi-layer penetration acceleration signals [26]. Figure 1a shows the measured acceleration curve of a 125 mm caliber shell penetrating a three-layer target. In contrast, Figure 1b shows the measured acceleration curve of a rocket sled penetrating a nine-layer target.

**Figure 2:**
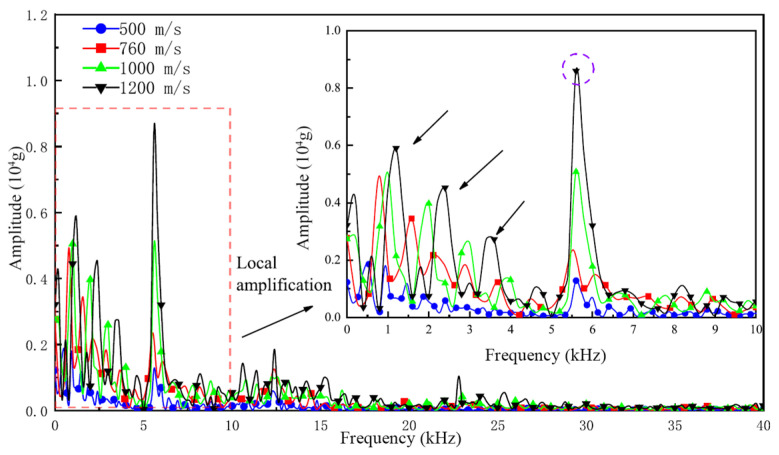
High-speed transient complex multi-layer acceleration signal frequency-domain characteristics [8]. The arrows indicate that the frequency distribution of the missile body acceleration signal is primarily concentrated in the 0–5 kHz range. The dotted circle highlights a significant resonance peak in the 5 kHz–10 kHz range.

The frequency-domain characteristics of a signal are fundamental properties. Multi-layer penetration acceleration signals contain components across multiple frequency bands, which reflect the attenuation effects of different layers and materials on the signal. The frequency distribution reveals essential characteristics of the signal transmission process, such as attenuation and frequency changes, making spectral analysis an effective tool for understanding signal properties. Liu et al. [8] presented the frequency-domain plots of acceleration signals during penetration at different initial velocities (as shown in Figure 2). The study indicated that the frequency-domain distributions are generally similar across different initial velocities, with the frequencies of the projectile penetration acceleration signals primarily concentrated in the 0–5 kHz range. When the frequencies exceed 5 kHz, the signal exhibits a single large peak and multiple smaller peaks. The larger peaks correspond to resonance frequencies, while the smaller peaks reflect other interfering components.

### 2.2. Analysis of the Causes of Complex Noise

Complex noise arises from electromagnetic interference affecting the accelerometer and mechanical oscillation interference caused by the internal structure of high-speed moving bodies.

#### 2.2.1. Accelerometer Electromagnetic Noise Analysis

**Figure 3:**
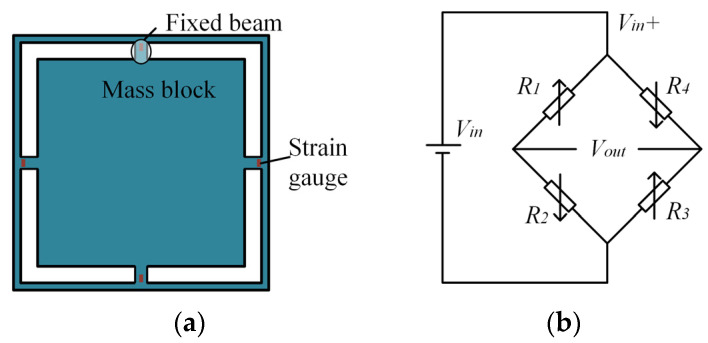
Accelerometer-sensitive chip structure and Wheatstone bridge structure: (**a**) accelerometer-sensitive chip structure; and (**b**) Wheatstone bridge structure.

The classic Liang Island structure is used as the accelerometer design for analysis, featuring symmetrical four-sided fixed beams around the perimeter and a mass block at the center, as shown in Figure 3a. Under the influence of external acceleration *a*, the mass block *m* inside the accelerometer converts the acceleration into inertial force *F* = *ma*, causing the four beams to generate bending moments *M* and stresses *σ*. The maximum stress *σ_max_* is then obtained by calculating the stress based on the bending moment:

(1) $$\sigma_{max}=\frac{3maL}{2bh^{2}}$$

Here, *L* is the length of a single fixed beam, *b* is the width, and *h* is the thickness of the fixed beam.

If the strain gauge is placed at the point of maximum deformation on the fixed beam, the relative change in resistance can be determined based on the piezoresistive effect:

(2) $$\frac{\Delta R}{R}=\frac{\Delta L}{L}+\frac{\Delta \rho }{\rho }-2\frac{\Delta r}{r}$$

Here, Δ*L*/*L* = *ε* represents the axial relative deformation of the strain gauge, also known as longitudinal strain; Δ*r*/*r* = *−vε* (where *v* is the Poisson’s ratio of the strain gauge material) represents the radial relative deformation, also known as transverse strain; and Δ*ρ*/*ρ* = *πσ* (where *π* is the piezoresistive coefficient) represents the relative change in the resistivity of the strain gauge.

For semiconductor materials, Δ*ρ*/*ρ* ≫ (1 + 2*v*) *E* (where *E* is the elastic modulus of the material). Therefore, it can be assumed that the change in resistance of the strain gauge is primarily due to changes in resistivity, i.e., Δ*R*/*R* ≈ Δ*ρ*/*ρ* = *πσ*.

The sensor structure utilizes the Wheatstone bridge principle (Figure 3b), which precisely measures the strain and acceleration through changes in the resistance of four piezoresistors. Ideally, the four pressure-sensitive resistors in a Wheatstone bridge have the same resistance value. When at rest:

(3) $$V_{out0}=\frac{R_{1}R_{3}-R_{2}R_{4}}{\left(R_{1}+R_{2}\right)\left(R_{3}+R_{4}\right)}V_{in}=0$$

Under the influence of the acceleration force, the beam deforms, causing the resistance of the pressure-sensitive resistors to change (Δ*R*), which in turn leads to a change in the output voltage of the Wheatstone bridge. The change in resistance (Δ*R*) is the same for each pressure-sensitive resistor. When *R*_1_ and *R*_3_ increase, *R*_2_ and *R*_4_ decrease, resulting in:

(4) $$V_{out}=\frac{\left(R_{1}+\Delta R)(R_{3}+\Delta R)-(R_{2}-\Delta R)(R_{4}-\Delta R\right)}{\left(R_{1}+R_{2}\right)\left(R_{3}+R_{4}\right)}V_{in}$$

When pressure-sensitive resistors are placed in areas of the beams experiencing greater deformation, the above equation can be simplified to:

(5) $$V_{out}=\frac{\Delta R}{R}V_{in}=\pi \sigma V_{in}=\frac{\pi 3mLV_{in}}{2bh^{2}}\cdot a$$

In high-speed motion controller systems, accelerometers require conditioning circuits to process signals. However, during this process, electromagnetic interference noise inevitably couples into the signal path, primarily categorized into two types: electromagnetic radiation interference and conducted interference. Electromagnetic radiation interference (*V_noise-out_*) originates from the external environment and enters high-impedance nodes through spatial coupling. In contrast, conducted interference (*V_noise-in_*) originates from within the system, caused by power supply oscillations due to high-impact loads and crosstalk introduced by capacitive coupling, which affects the acceleration signal. These interferences can degrade signal integrity and potentially lead to false triggers or data loss during the “counting” process.

(6) $$V_{out\_total}=\underset{acceleration}{\underbrace{K\cdot a}}+\underset{interferences}{\underbrace{V_{noise-out}+V_{noise-in}}}$$

where *K* is the linear proportional coefficient.

#### 2.2.2. Accelerometer Mechanical Oscillation Noise Analysis

The signal detected by the accelerometer’s sensitive chip is transmitted by a high-speed moving body, controller, accelerometer, and sensitive chip.

**Figure 4:**
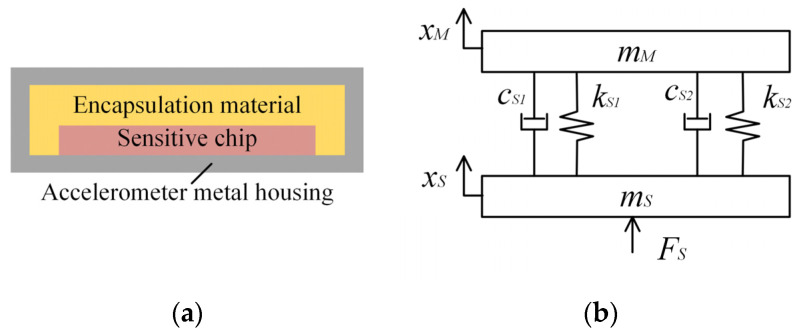
Accelerometer-sensitive chip system: (**a**) structural diagram; and (**b**) dynamic transfer model.

Figure 4a shows a schematic diagram of the packaged accelerometer-sensitive chip structure, which consists of the accelerometer metal housing, encapsulation material, and sensitive chip. The accelerometer-sensitive chip has two transmission paths: one directly from the metal housing to the sensitive chip, and the other through the encapsulation material to the sensitive chip. Based on these contact methods, a dynamic transmission model for the accelerometer-sensitive chip system is established (Figure 4b). In this model, *m_M_* and *m_S_* represent the masses of the sensitive chip (mass block) and the accelerometer metal housing, respectively. *x_M_* and *x_S_* denote the displacement vectors of the sensitive chip and the accelerometer metal housing, respectively. *k_S_*_1_ and *c_S_*_1_ are the equivalent stiffness coefficient and equivalent damping coefficient for the direct contact between the sensitive chip and the metal housing, respectively. *k_S_*_2_ and *c_S_*_2_ are the equivalent stiffness coefficient and equivalent damping coefficient for the encapsulation material, respectively.

The transfer function of the accelerometer-sensitive chip system dynamics is:

(7) $$\left\{\begin{array}{l} m_{S}{\ddot{x}}_{S}-\left(k_{S1}+k_{S2}\right)\left(x_{M}-x_{S}\right)-\left(c_{S1}+c_{S2}\right)\left({\dot{x}}_{M}-{\dot{x}}_{S}\right)=F_{S}\\ m_{M}{\ddot{x}}_{M}+\left(k_{S1}+k_{S2}\right)\left(x_{M}-x_{S}\right)+\left(c_{S1}+c_{S2}\right)\left({\dot{x}}_{M}-{\dot{x}}_{S}\right)=0 \end{array}\right.$$

where *F_S_* represents the external force on the metal casing of the accelerometer.

**Figure 5:**
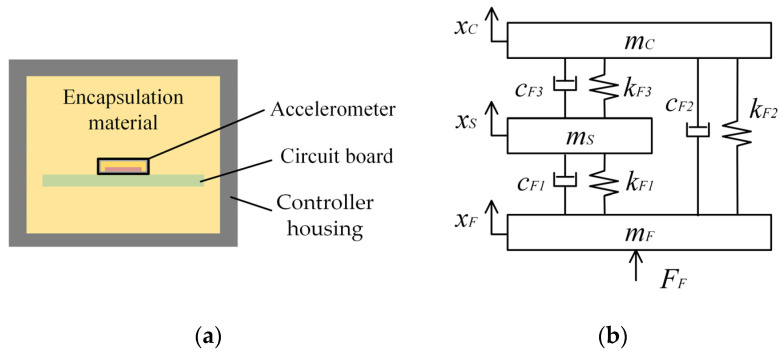
Controller–accelerometer system: (**a**) structural diagram; and (**b**) dynamic transfer model.

Figure 5a shows a schematic diagram of the controller–accelerometer structure, which consists of encapsulation material, an accelerometer, a circuit board, and a controller housing. The controller–accelerometer system has two transmission paths: one directly from the controller housing through the encapsulation material to the accelerometer, and the other from the controller housing through the encapsulation material to the circuit board, which then transmits to the accelerometer. Based on these contact configurations, a dynamic transmission model for the controller–accelerometer system is established (Figure 5b). In this model, *m_C_* and *m_F_* represent the masses of the circuit board and controller housing, respectively. *x_C_* and *x_F_* represent the displacement vectors of the circuit board and controller housing, respectively. *k_F_*_1_ and *c_F_*_1_ denote the equivalent stiffness coefficient and equivalent damping coefficient of the encapsulation material between the accelerometer and the controller housing, respectively. *k_F_*_2_ and *c_F_*_2_ denote the equivalent stiffness coefficient and equivalent damping coefficient of the connection between the accelerometer and circuit board. *k_F3_* and *c_F3_* denote the equivalent stiffness coefficient and equivalent damping coefficient of the encapsulation material between the circuit board and the accelerometer.

The transfer function of the controller–accelerometer system dynamics is:

(8) $$\left\{\begin{array}{l} m_{F}{\ddot{x}}_{F}-c_{F1}({\dot{x}}_{S}-{\dot{x}}_{F})-k_{F1}(x_{S}-x_{F})-c_{F2}({\dot{x}}_{C}-{\dot{x}}_{F})-k_{F2}(x_{C}-x_{F})=F_{F}\\ m_{S}{\ddot{x}}_{S}-c_{F3}({\dot{x}}_{C}-{\dot{x}}_{S})-k_{F3}(x_{C}-x_{S})+c_{F1}({\dot{x}}_{S}-{\dot{x}}_{F})+k_{F1}(x_{S}-x_{F})=0\\ m_{C}{\ddot{x}}_{C}+c_{F3}({\dot{x}}_{C}-{\dot{x}}_{S})+k_{F3}(x_{C}-x_{S})+c_{F2}({\dot{x}}_{C}-{\dot{x}}_{F})+k_{F2}(x_{C}-x_{F})=0 \end{array}\right.$$

where *F_F_* represents the external force on the controller housing.

Similarly, a model of the high-speed moving-body–controller system can be constructed, and an ideal transmission path from the high-speed moving body to the sensitive chip can be established. However, during actual transmission, due to complex factors such as nonlinear spring-damping characteristics and the periodic opening and closing of structural gaps, the system exhibits significant nonlinear dynamic responses. This nonlinear behavior interferes with the accelerometer’s precise sensing of the acceleration signal, introducing additional nonlinear noise, which in turn affects the reliable detection of the sensitive chip and system stability.

### 2.3. Common Signal Noise Reduction Methods and Limitations

Multi-layer acceleration signals are often accompanied by noise. As analyzed in the previous section, the noise mainly originates from electromagnetic interference and mechanical oscillation interference affecting the sensor. These noises significantly disrupt the effective features of the signal, thereby affecting the accuracy of the signal recognition and analysis. Therefore, selecting appropriate noise reduction methods is crucial in signal processing. Commonly used noise reduction techniques include EEMD and wavelet threshold denoising.

#### 2.3.1. EEMD

Empirical mode decomposition (EMD) is an adaptive nonlinear/non-stationary signal-processing method proposed by Huang et al. (1998) [15]. Its core principle is to decompose a signal into a finite number of intrinsic mode functions (IMFs) and a residual term. IMFs must satisfy the condition that the difference between the number of zero crossings and extrema is ≤1, and at any given time, the mean value defined by the envelope of the extrema is zero. The result is expressed as:

(9) $$x(t)=\sum_{i=1}^{n}{\mathrm{IMF}}_{i}(t)+r(t)$$

EMD is commonly used for extracting signal features, with the resulting components serving as input for subsequent analysis methods to complete complex tasks. Compared to methods such as the Fourier transform and wavelet decomposition, EMD is more intuitive and straightforward. However, due to the influence of the time-scale characteristics of the original signal, EMD suffers from modal-aliasing issues.

EEMD is an improvement on the traditional EMD method, designed to address EMD’s sensitivity to noise. Proposed by Wu and Huang in 2009 [16], EEMD involves adding white noise to the original signal, performing EMD decomposition on the signal after multiple noise additions, and finally, averaging the results of these decompositions. This effectively reduces the modal overlap and improves the stability and noise resistance of the decomposition.

The process of the EEMD signal decomposition algorithm is as follows:

1. A Gaussian white noise sequence *ε_i_*(*t*) is added to the original signal *x*(*t*) to generate a noisy signal:

(10) $$x_{i}\left(t\right)=x\left(t\right)+\varepsilon_{i}\left(t\right)\;(i=1,2,\ldots N)$$

2. EMD is independently performed on each *x_i_*(*t*) to obtain the IMF set *c_ik_*(*t*) (where *k* is the decomposition order, representing the *k*-th order IMF component, and the total decomposition order is *K*), as well as the residual term *r_i_*(*t*).
3. Integrated averaging is performed on the IMF components of the same order and the residual term *r_i_*(*t*) to offset the effect of the added Gaussian white noise.

(11) $${\bar{c}}_{k}\left(t\right)=\frac{1}{N}\overset{N}{\underset{i=1}{\sum }}c_{ik}\left(t\right)$$

(12) $$r\left(t\right)=\frac{1}{N}\overset{N}{\underset{i=1}{\sum }}r_{i}\left(t\right)$$

EEMD achieves noise reduction by discarding low-order high-frequency IMF components. However, these discarded low-order high-frequency IMF components may contain important transient features. Therefore, it is necessary to retain all the IMF components and perform secondary noise reduction on the low-order high-frequency noisy IMF components.

#### 2.3.2. Wavelet Threshold Denoising

**Figure 6:**
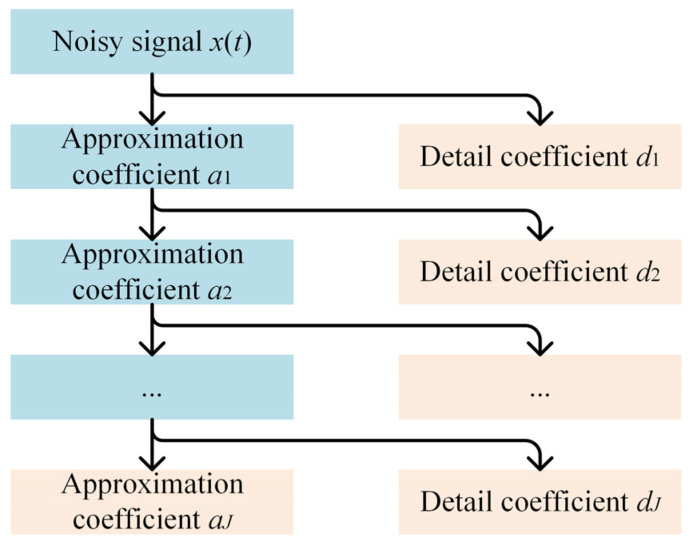
Wavelet decomposition process.

Wavelet threshold denoising is a preprocessing method based on the distribution characteristics of signals and noise in the wavelet domain. Its core principle is that the energy of a signal is concentrated in a small number of significant wavelet coefficients, mainly in the low-frequency approximation coefficients. The energy of noise is spread across a large number of small-amplitude high-frequency detail coefficients. By applying threshold quantization to the detail coefficients, noise can be effectively suppressed while retaining the main features of the signal. The workflow of the wavelet threshold denoising algorithm is as follows:

1. Perform *J*-layer discrete wavelet transform (DWT) on the noisy signal *x*(*t*):

(13) $$\left\{a_{J},d_{J},d_{J-1},\cdots ,d_{1}\right\}=\mathrm{DWT}\left(x\left(t\right)\right)$$

where *a_J_* is the approximation coefficient for the *J*-th layer (low-frequency component), and *d_j_* (*j* = 1, 2, …, *J*) are the detail coefficients for each layer (high-frequency components). The decomposition process is shown in Figure 6.

2. Apply threshold processing to the coefficients obtained from the decomposition. The approximation coefficients, which carry the main signal features, are retained unchanged. The detail coefficients are quantized according to the threshold function:

(14) d^j=ηλdj

3. Perform the discrete wavelet inverse transform using the processed coefficients.

(15) xdt=IDWTaJ,d^1,d^2,…, d^J

The output *x_d_*(*t*) is the noise-reduced signal.

The wavelet threshold denoising algorithm relies on threshold selection and fixed threshold functions, but it has limitations. Threshold selection is influenced by the noise intensity and signal characteristics, which can result in insufficient noise suppression or the loss of signal details. Additionally, hard and soft thresholds may introduce reconstruction errors or excessive smoothing in certain scenarios, failing to balance noise removal with signal preservation. Therefore, a single wavelet threshold denoising method struggles to achieve optimal results in complex noise environments and requires integration with other methods for improvement.

## 3. Methods

In the previous section, the features of multi-layer continuous acceleration signals and the sources of their complex noise were analyzed, while also addressing the limitations of existing denoising methods (EEMD and single wavelet threshold denoising) in preserving signal features and removing complex noise. To address these issues, this paper proposes a method based on sample entropy modal partitioning combined with ensemble empirical mode decomposition and improved wavelet threshold denoising (EEMD-SE-IWTD). This method effectively processes high-speed transient complex feature acceleration signals during the penetration process, enhancing the denoising performance while preserving the key features of the signal.

### 3.1. Systematic Process

**Figure 7:**
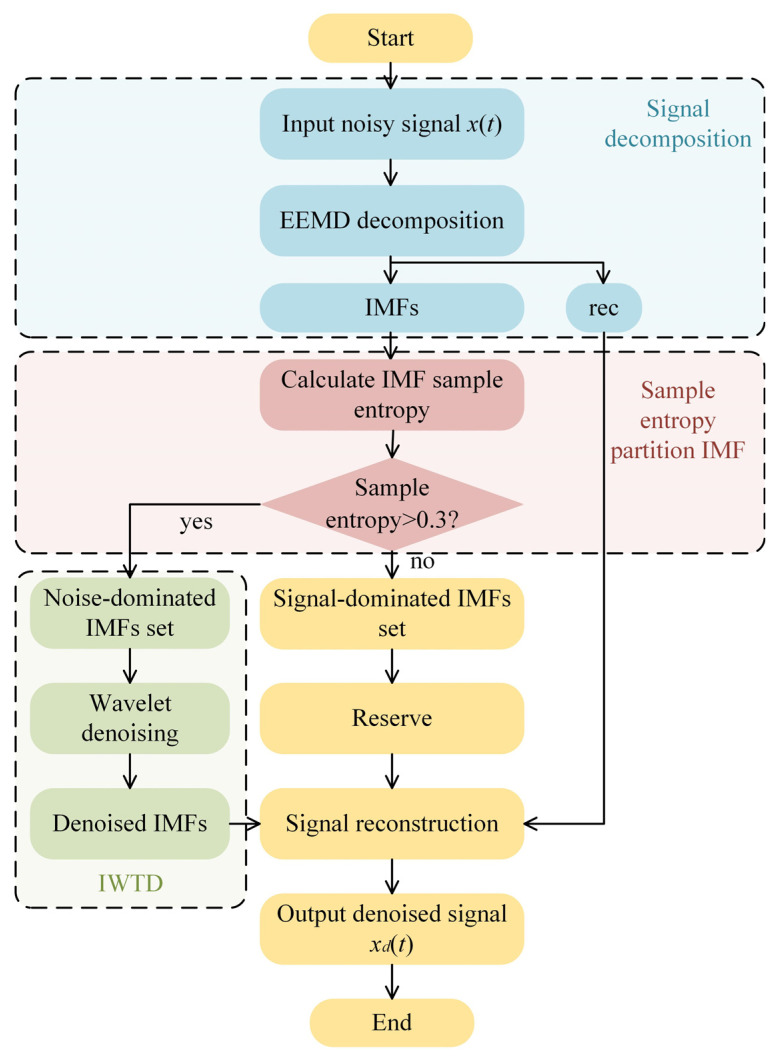
EEMD-SE-IWTD noise reduction process.

The EEMD-SE-IWTD method decomposes signals using EEMD, separates the noise and effective signal IMF components based on sample entropy thresholds, and incorporates improved wavelet threshold denoising (IWTD) technology to achieve more efficient denoising performance and better signal feature retention. The specific process is illustrated in Figure 7.

The noisy signal *x*(*t*) is decomposed into IMF components, which are arranged in descending order of frequency using EEMD. Since the noise levels of different IMF components vary, this difference is crucial for subsequent processing. For high-speed transient acceleration signals with complex features and multiple layers, noise concentrates in high-frequency signals and is primarily distributed in lower-order IMF components. To distinguish between complex noise-dominated components and effective transient feature-dominated components, it is necessary to identify a key component for division. For this purpose, sample entropy is introduced as the basis for partition. Sample entropy, proposed by Richman et al. [27], is a measure of the complexity and irregularity of a time series. Its main advantage lies in the fact that the calculation results are not significantly affected by the sequence length *N*, and the dimensions *m* and tolerance *r* have a positive correlation with the entropy value, ensuring the stability of the results.

The principle of sample entropy calculation is as follows. Given a time series {*x*(*n*)}= *x*(1), *x*(1), …, *x*(*N*):

1. Construct vectors:

(16) $$X_{m}\left(i\right)=\left\{x\left(i\right),x\left(i+1\right),\cdots ,x\left(i+m-1\right)\right\}\;(1\leq i\leq N-m+1)$$

2. Define vector distance:

(17) $$d\left[X_{m}\left(i\right),X_{m}\left(j\right)\right]=\max_{k=0,\cdots ,m-1}\left(\left|x\left(i+k\right)-x\left(j+k\right)\right|\right)$$

3. Calculate statistical similarity.

For a given *X_m_*(*i*), count the number of *X_m_*(*i*) and *X_m_*(*j*) whose distance is ≤*r* (1 ≤ *j* ≤ *N-m*, *j* ≠ *i*) and denote this as *B_i_*. For 1 ≤ *i* ≤ *N − m*, define:

(18) $$B_{i}^{m}\left(r\right)=\frac{1}{N-m-1}B_{i}$$

The global similarity probability is defined as:

(19) $$B^{m}\left(r\right)=\frac{1}{N-m}\sum_{i=1}^{N-m}B_{i}^{m}\left(r\right)$$

4. Increase the dimension to *m* + 1, and calculate *B^m^*^+1^(*r*) in the same way.
5. The sample entropy formula is as follows:

(20) $$SampEn\left(m,r,N\right)=-\ln\left[\frac{B^{m+1}\left(r\right)}{B^{m}\left(r\right)}\right]$$

According to the literature [28], the value of *m* is generally chosen as 1 or 2, and *r* = (0.1–0.25) *σ* (where *σ* is the standard deviation of the sequence). In this paper, *m* = 2 and *r* = 0.25*σ* are selected.

The sample entropy is calculated for all the IMF components. IMF components with entropy values greater than 0.3 are in the high-entropy region, which is dominated by complex noise. These components exhibit high complexity while still retaining some signal features, indicating the need for further enhancement through wavelet threshold denoising. For IMF components in the high-entropy region, the wavelet threshold processing is performed using the db8 wavelet basis. The number of wavelet decomposition levels is set to five. The threshold function selected for the improved wavelet threshold denoising method is the soft threshold. The improved Sqtwolog threshold principle is selected for the wavelet threshold denoising, and the corresponding calculation formula is as follows:

(21) $$T=f_{w}\sigma_{w}\sqrt{2\log\left(N\right)}$$

Here, *σ_w_* represents the standard deviation of noise in the wavelet domain, calculated as *σ_w_* = Median(|*d_j_*|)/0.6745. *N* denotes the number of sample points. *f_w_* is the adaptive coefficient based on the improved threshold principle using wavelet decomposition properties, calculated as follows:

(22) $$f_{w}=1.5+0.5(level-3)/level$$

where *level* represents the number of wavelet decomposition levels. *f_w_* = 1 represents the standard Sqtwolog threshold, and when the coefficient is increased to *f_w_* = 1.5, the overall noise reduction intensity increases, allowing for more effective noise removal. Building on this, the coefficient is further adjusted to change according to the number of decomposition levels adaptively. At low decomposition levels, the coefficient is kept small, ensuring that the structure of the relatively low-frequency signals in the lower approximation coefficients is preserved, thus reducing excessive noise suppression. At high decomposition levels, the threshold coefficient increases, effectively enhancing high-frequency noise suppression in the higher-level approximation coefficients. Through this adaptive adjustment, more fine-grained noise reduction can be achieved across different frequency ranges, thereby preserving signal characteristics while enhancing the noise removal efficiency. The effectiveness of this adaptive coefficient is experimentally validated in Section 4.3.

IMF components with entropy values less than or equal to 0.3 are classified as being in the low-entropy region, which preserves the low-frequency characteristics of the signal and is left unprocessed.

Finally, the denoised high-entropy-region IMF components, the unprocessed low-entropy-region IMF components, and the residual term are combined to reconstruct the denoised signal.

### 3.2. Experimental Design

#### 3.2.1. Signal Source and Design

To compare the noise reduction effects of traditional methods with the EEMD-SE-IWTD method, this study simulates multi-layer through-acceleration signals containing complex interference noise. The specific process is as follows. Clean multiple impact signals were obtained from penetration experiments using the existing experimental apparatus. Gaussian white noise was then introduced into the clean impact signals to simulate multi-layer penetration acceleration signals with high-speed transient complex features in actual environments.

- Multiple penetration experiments

**Figure 8:**
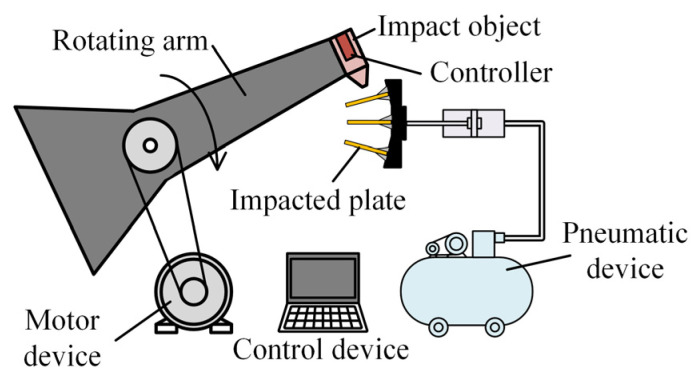
Overall schematic diagram of the multiple-pass experimental apparatus.

**Figure 9:**
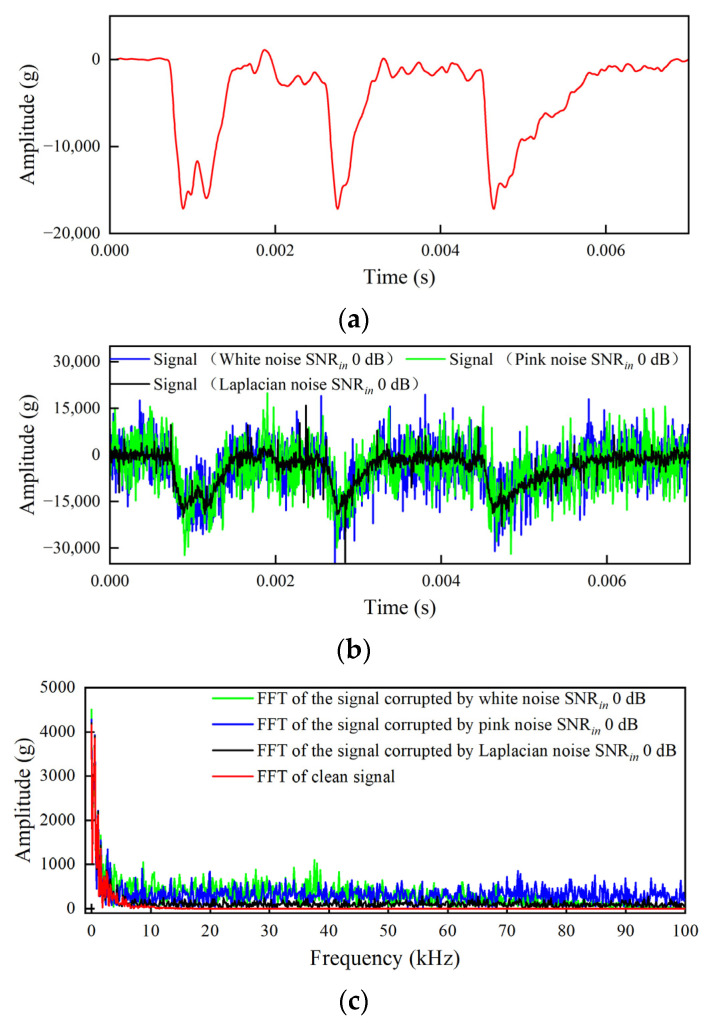
Time-domain and frequency-domain diagrams of the simulation results for multi-layer penetration acceleration signals: (**a**) clean multiple impact signals; (**b**) simulated results of multi-layer through-acceleration signals with SNR*_in_* 0 dB with each type of noise superimposed; and (**c**) frequency-domain plots of clean and noisy signals.

This paper obtains clean multiple impact signal data through multiple penetration experiments. The experiments are conducted using a self-built apparatus designed for multi-layer penetration to collect acceleration signals from the impacted plate. As shown in Figure 8, the apparatus consists of an electric drive, a rotating arm, an impact object, a controller, an impacted plate, a pneumatic device, and a control device. The electric drive rotates the impact object via the rotating arm, while the pneumatic device controls the feeding and retraction of the impacted plate. The control device adjusts the rotation speed of the rotating arm and the feeding motion of the impacted plate, enabling directed penetration of the impact object. The controller is fixed to the impact object with threads and includes an accelerometer to measure the acceleration signals of the impact object as it penetrates multiple layers of the impacted plate. The typical experimental signals obtained are shown in Figure 9a, which demonstrate clean multiple impact signals.

- Simulation of high-speed transient complex feature multi-layer penetration acceleration signals

In actual penetration tests, the multi-layer penetration acceleration signals collected and recovered are essentially high-speed transient complex feature multi-layer penetration acceleration signals, as shown in Figure 1b. These signals result from the superposition of clean rigid body penetration acceleration signals *x_c_*(*t*) and noise interference *x_b_*(*t*) [29].

(23) $$x\left(t\right)=x_{c}\;\left(t\right)+x_{b}\left(t\right)$$

To simulate the complex features of high-speed transient multi-layer acceleration signals, various types of noise *x_b_*(*t*), including controllable Gaussian white noise, pink noise, and Laplace noise, are added to the multiple impact signals obtained in the test. The noise power is calculated based on the specified input signal-to-noise ratio (SNR*_in_*), which is defined as follows:

(24) $${\mathrm{SNR}}_{in}=10\log_{10}\left(\frac{P_{c}}{P_{b}}\right)$$

where the signal power *P_c_* is given by $P_{c}=1/N\cdot \sum_{i=1}^{N}x_{c}^{2}(i)$, and the noise power $P_{b}$ is given by $P_{b}=1/N\cdot \sum_{i=1}^{N}x_{b}^{2}(i)$. Given the SNR*_in_* of the target, the required noise power can be calculated to generate the noise signal. The formula for calculating the power of white noise is as follows:

(25) $$P_{b}=\frac{P_{c}}{10^{{\mathrm{SNR}}_{in}/10}}$$

The power calculations for pink and Laplace noise are similar to those for white noise, but their characteristics and power distributions differ. Pink noise typically exhibits frequency component correlation, while Laplace noise has a stronger spiking characteristic. To generate these noise signals, the power of each type can be modeled by adjusting the parameters associated with the noise type.

Overlay noise with SNR*_in_* values of −10 dB, −5 dB, 0 dB, 5 dB, and 10 dB onto the clean multiple impact signals (sampling rate of 200 kHz, 1400 points, and a waveform interval of 2 ms) to produce noise-containing signals with different initial SNR*_in_* values.

Figure 9 shows the time-domain and frequency-domain plots of the simulation results for clean impact signals and multi-layer through-acceleration signals with a superimposed SNR*_in_* of 0 dB. The time-domain waveform comparison (Figure 9b) demonstrates the destructive effect of additive noise on the original clean impact signal when the SNR*_in_* is 0 dB. Compared to the relatively smooth and well-defined waveform of the clean signal (with an amplitude range from −20,000 g to 0 g), the noise-contaminated signal (SNR*_in_* 0 dB signal) exhibits severe random oscillations, with its amplitude range significantly expanded from −30,000 g to +15,000 g. The key transient features of the original clean impact signal, including peaks, zero crossings, and waveform contours, are almost completely overwhelmed by high-intensity noise. This indicates that under such adverse signal-to-noise ratio conditions (SNR*_in_* = 0 dB), it is challenging to extract or identify the features of the original clean impact signal directly from the time-domain waveform.

Frequency-domain analysis (FFT results, Figure 9c) further quantifies the interference of noise at an SNR*_in_* of 0 dB. The spectrum of the clean signal is concentrated within 5 kHz and is relatively smooth outside of 5 kHz. In contrast, the spectrum of the 0 dB SNR*_in_* signal exhibits the following key characteristics. The background noise floor across the entire frequency band is significantly elevated (up to 500 g), indicating that noise energy is distributed across a wide bandwidth. The white-noise-containing signals are the most homogeneous across the full range, with prominent spectral peaks in the entire range (500–1000 g). The pink-noise-containing signals show a gradual decrease in noise peaks as frequency increases. The overall enhancement of Laplace noise is low.

The method described above simulates high-speed transient complex features multi-layer acceleration signals, providing a more effective signal source and evaluation basis for noise reduction performance.

#### 3.2.2. Comparison and Selection of Noise Reduction Methods

Seven noise reduction methods were compared with the method proposed in this paper. Among them are four similar variants of noise reduction methods related to the methods in this paper, as well as three advanced noise reduction methods.

The similar variants of noise reduction methods include the ensemble empirical mode decomposition method based on sample entropy mode partitioning (EEMD-SE), the classic wavelet threshold denoising (CWTD) method, and the improved wavelet threshold denoising (IWTD) method. Additionally, the ensemble empirical mode decomposition and classic wavelet threshold denoising (EEMD-SE-CWTD) method, based on sample entropy mode partitioning, was also considered. The proposed method is the ensemble empirical mode decomposition and improved wavelet threshold denoising (EEMD-SE-IWTD) method, also based on sample entropy mode partitioning. The parameters are shown in Table 1.

The selection of these four methods includes both classical noise reduction techniques (such as CWTD and EEMD-SE) and improved noise reduction methods (such as IWTD and EEMD-SE-CWTD). By comparing these methods, this paper demonstrates the effect of different improvements on noise reduction performance. For instance, by adding CWTD to the EEMD-SE method and enhancing EEMD-SE-CWTD to IWTD, each step of the improvement contributes unique performance changes. It is also compared with both the CWTD method and the IWTD method. These comparisons not only highlight the innovative aspects of the EEMD-SE-IWTD method relative to traditional methods but also demonstrate the potential of improved methods in practical applications.

In addition to similar method variants, three advanced noise reduction methods with broader applications and outstanding performance are introduced for comparison. The methods selected for comparison include the following. (1) Complete ensemble empirical mode decomposition with adaptive noise (CEEMDAN), an improved algorithm of EEMD, which offers better modal completeness and decomposition adaptability. (2) Variational mode decomposition (VMD) [30], which utilizes a variational framework for adaptive modal decomposition, providing strong theoretical rigor and operational stability. (3) Synchrosqueezed transform, based on the short-time Fourier transform (FSST) [31,32], which excels in energy aggregation in time–frequency characterization and significantly improves the accuracy of time–frequency resolution.

Selecting these seven methods for comparison provides a comprehensive presentation of the advantages of the EEMD-SE-IWTD method across different noise reduction techniques, fully demonstrating its innovation and practical value. Through this multi-dimensional comparative analysis, this paper spans classical noise reduction methods and their variants to advanced noise reduction methods, ensuring the comprehensiveness and scientific rigor of the study.

#### 3.2.3. Evaluation Methods

To comprehensively evaluate and quantify the performance of various noise reduction methods, the output signal-to-noise ratio (SNR*_out_*), output root mean square error (RMSE*_out_*), and output correlation coefficient (CC*_out_*) are used as fundamental evaluation indicators. Furthermore, through standardized processing and weighted fusion, a comprehensive evaluation score (CES) is constructed to achieve multi-dimensional quantification of the noise reduction performance.

The output signal-to-noise ratio (SNR*_out_*) reflects the relative intensity of residual noise in the noise reduction signal. The higher the value, the better the noise reduction effect:

(26) $${\mathrm{SNR}}_{out}=10\log_{10}\left(\frac{\sum_{i=1}^{N}x_{c}{\left(i\right)}^{2}}{\sum_{i=1}^{N}{\left[x_{d}(i)-x_{c}(i)\right]}^{2}}\right)$$

where *x_c_*(*i*) is the *i*-th original signal value, *x_d_*(*i*) is the *i*-th denoised signal value, and *N* is the total number of signal sample points.

The output root mean square error (RMSE*_out_*) characterizes the average deviation between the denoised signal and the original signal. The lower the value, the higher the waveform restoration accuracy.

(27) $${\mathrm{RMSE}}_{out}=\sqrt{\frac{1}{N}\sum_{i=1}^{N}{\left[x_{d}(i)-x_{c}(i)\right]}^{2}}$$

The output correlation coefficient (CC*_out_*) evaluates the linear correlation between two sets of signals. The closer the value is to 1, the more complete the feature structure that is retained.

(28) $${\mathrm{CC}}_{out}=\frac{\sum_{i=1}^{N}\left(x_{c}(i)-{\bar{x}}_{c}\right)(x_{d}(i)-{\bar{x}}_{d})}{\sqrt{\sum_{i=1}^{N}{\left(x_{c}(i)-{\bar{x}}_{c}\right)}^{2}}\sqrt{\sum_{i=1}^{N}{\left(x_{d}(i)-{\bar{x}}_{d}\right)}^{2}}}$$

where ${\overset{¯}{x}}_{c}$ and ${\overset{¯}{x}}_{d}$ represent the means of the original clean signal and the denoised signal, respectively.

## 4. Results

### 4.1. Analysis of Experimental Results

**Figure 10:**
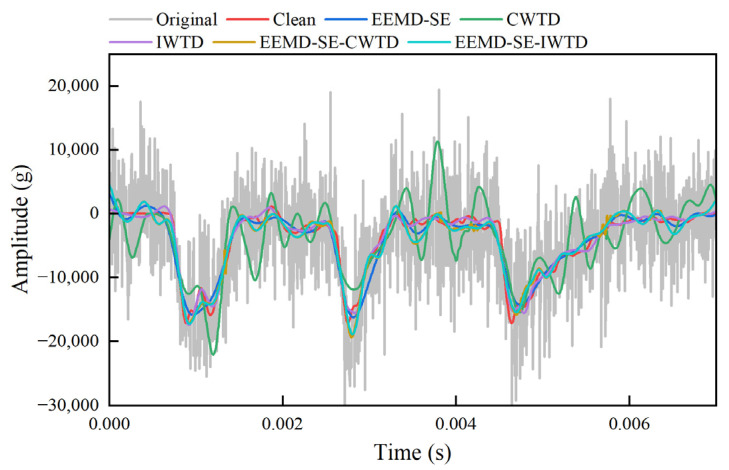
Clean signal, generated signal, EEMD-SE-IWTD noise reduction result, and similar variants noise reduction results curves for different noise reduction methods.

The generated signal with a 0 dB SNR*_in_* of white noise is used as an example to analyze the experimental effect. Figure 10 illustrates the result curves for the clean signal, the generated signal (with 0 dB SNR*_in_* white noise), the EEMD-SE-IWTD noise reduction result, and the similar variants of noise reduction results (EEMD-SE, CWTD, IWTD, and EEMD-SE-CWTD).

The analysis reveals that the noise reduction outcomes of the EEMD-SE method are excessively smooth, with only one waveform observed at each of the three peaks. This fails to accurately represent the fluctuations of the original clean signal at those peaks. The CWTD method overlooks signal non-stationarity, leading to substantial noise estimation errors and jitter throughout the entire period. While the IWTD method effectively suppresses high-frequency noise, it causes amplitude decay near the peaks at 0.0025 s and 0.0045 s, indicating the unintended removal of useful high-frequency components. The EEMD-SE-CWTD method, compared to the EEMD-SE and CWTD methods, suppresses noise without introducing excessive smoothness but still retains high-frequency spikes at 0.0012 s, 0.004 s, and 0.0058 s. In contrast, the method proposed in this paper (EEMD-SE-IWTD) shows no high-frequency spikes throughout the entire period and closely matches the clean signal features at all three peaks.

**Figure 11:**
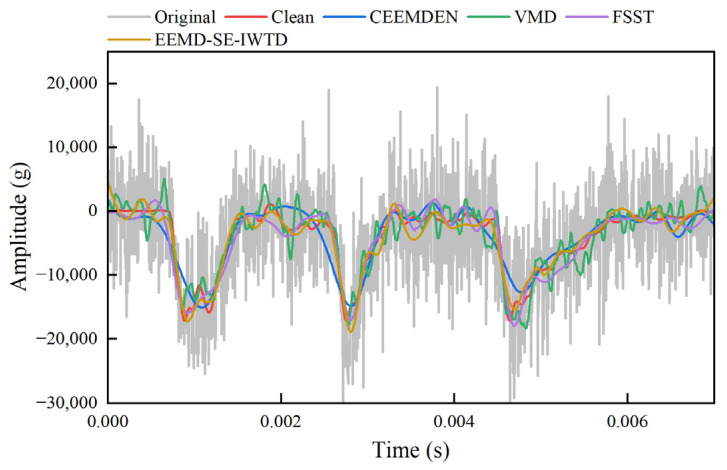
Clean signal, generated signal, EEMD-SE-IWTD noise reduction result, and advanced noise reduction results curves for different noise reduction methods.

Figure 11 illustrates the result curves for the clean signal, the generated signal (0 dB SNR*_in_* white noise), the EEMD-SE-IWTD noise reduction result, and the advanced noise reduction results (CEEMDAN, VMD, and FSST).

It is observed that the noise reduction results of the CEEMDAN and FSST methods tend to smooth the signal, with some loss of transient features (especially at the wave crests). In contrast, the VMD method, after denoising, makes the noise-reduced signal less realistic and causes distortion, despite retaining more of the noise signal. On the other hand, the EEMD-SE-IWTD method proposed in this paper produces noise reduction results that are closest to the clean signal. This method avoids excessive smoothing and effectively reduces the noise component, demonstrating its optimal performance in addressing the noise reduction problem.

### 4.2. Verification of the Modal Partitioning Mechanism Based on Sample Entropy

An analog noisy signal with a 0 dB SNR*_in_* of white noise is used as an example to perform sample entropy (SE) calculations on the seven IMF components decomposed by EEMD.

Table 2 presents the SE values, center frequencies, physical significance, and processing methods for each IMF component. As indicated in the literature [4,5,6,7,8], the energy of overload signals is primarily distributed within the ≤5 kHz frequency band, which aligns with the frequency ranges of IMF5 (1.570 kHz), IMF6 (0.571 kHz), and IMF7 (0.285 kHz) in Table 2. This indicates that these IMF components correspond to the main components of impact overload. Their waveforms exhibit regularity, consistent with the features of the physical process, and are therefore retained. In contrast, the center frequencies of IMF1 to IMF4 are relatively high, ranging from 72.234 kHz to 5.139 kHz. These components contain high-frequency resonance noise from the sensor or oscillatory responses from the target–projectile dynamics. Their time-domain fluctuations are irregular, manifesting as random noise and oscillations, and thus, IWTD noise reduction processing is applied. 

**Figure 12:**
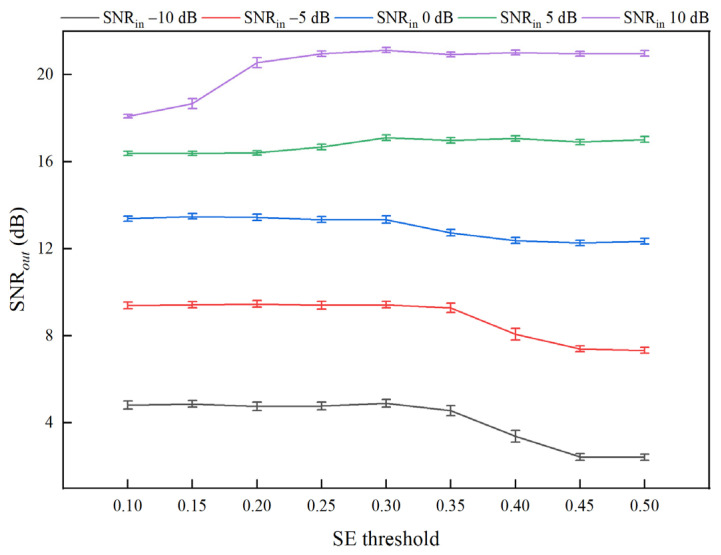
Sensitivity curves of SNR*_out_* with different SE values at different SNR*_in_*.

To analyze the sensitivity of the sample entropy threshold, a systematic parameter scan was conducted for the sample entropy threshold within the range of 0.10 to 0.50, with a step size of 0.05. EEMD-SE-IWTD denoising tests were performed under SNR*_in_* conditions of −10, −5, 0, 5, and 10 dB. For each condition, the SNR*_out_*, RMSE*_out_*, and correlation coefficient (CC*_out_*) were calculated, with 95% confidence intervals determined through 100 repeated experiments. Using white noise signals as an example, the denoising metrics for different SE values are presented in Table 3, and the denoising performance is illustrated in Figure 12.

Figure 12 illustrates the sensitivity curves of the SNR*_out_* with different values of SE under various SNR*_in_* conditions. Combined with the results in Table 3, it shows that when SE is in the range of 0.25 to 0.30, the SNR*_out_*, RMSE*_out_*, and CC*_out_* are all at optimal levels, with no significant performance differences. When SE ≥ 0.35, the performance significantly decreases under −10 dB, −5 dB, and 0 dB conditions. When SE ≤ 0.2, the performance decreases under 5 dB and 10 dB conditions. Considering the performance across all the conditions, SE = 0.30 demonstrates strong robustness under all the SNR*_in_* conditions, with negligible deviation from the optimal value. Therefore, SE = 0.30 is selected as the final threshold.

To summarize the findings, this paper demonstrates that the sample entropy threshold exhibits stable robustness within the SE = 0.25–0.30 range through parameter scanning and statistical testing. The SE value for distinguishing high-entropy and low-entropy regions is set to 0.30, effectively differentiating between noise and signal features, ensuring the retention of valid signals and the suppression of noise.

### 4.3. Validation of the Improved Threshold Principle for EEMD-SE-IWTD

To verify the advantages of the improved threshold principle adaptive coefficient method, the 0 dB SNR*_in_* with a white-noise-containing signal is used as an example. The IMF components from the EEMD method in this paper are subjected to IWTD noise reduction, with the IMF components having SE > 0.30. The threshold principle coefficients selected are *f_w_* = 1 (Sqtwolog threshold principle), *f_w_* = 1.5 (improved high-coefficient Sqtwolog threshold principle), and *f_w_* = 1.5 + 0.5(*level* − 3)/*level* (improved adaptive thresholding principle coefficients for Sqtwolog thresholding).

**Figure 13:**
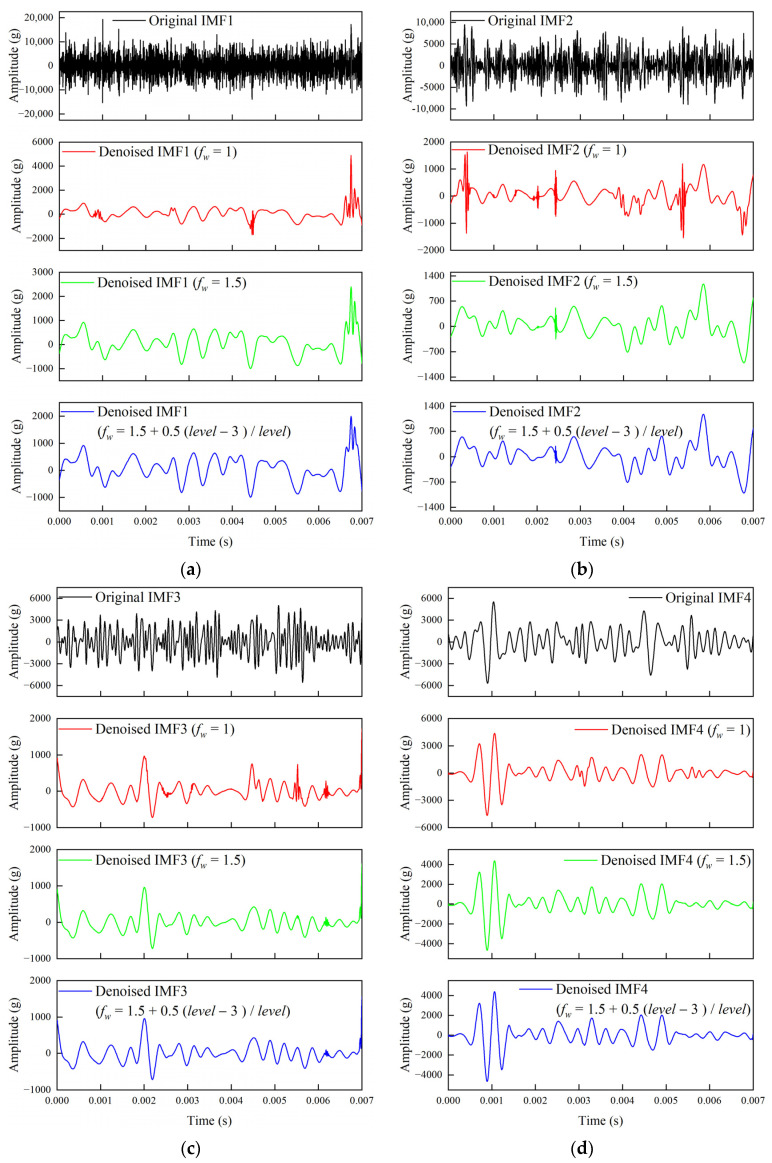
Wavelet thresholding noise reduction results with different thresholding principle improvement coefficients for IMF components with SE > 0.30: (**a**) IMF1 noise reduction results; (**b**) IMF2 noise reduction results; (**c**) IMF3 noise reduction results; and (**d**) IMF4 noise reduction results.

Figure 13 illustrates the noise reduction results of different threshold principle coefficients (*f_w_*) for the noise-containing IMF components. The results indicate that when *f_w_* = 1, burr oscillations are prevalent in most IMF components, especially in IMF1 and IMF2, where the burr oscillations are more pronounced, while fewer burr oscillations are observed in IMF3 and IMF4. When *f_w_* = 1.5, the noise reduction intensity increases, and burr oscillations are effectively suppressed in most components. However, burr oscillations remain at certain time points (at 0.0068 s for IMF1, 0.0023 s for IMF2, 0.0061 s and 0.007 s for IMF3). The overall waveform trend does not change significantly compared to when *f_w_* = 1. When *f_w_* = 1.5 + 0.5(*level* − 3)/*level*, burr oscillations are further reduced, and the signal trend remains stable.

For wavelet threshold noise reduction applied to high-frequency IMF components, low-level wavelet decomposition has a lower noise reduction intensity, which helps retain the effective low-frequency transient features of high-frequency IMF components in the low-level approximation coefficients. In contrast, higher-level wavelet decomposition has a stronger noise reduction intensity, effectively suppressing high-frequency noise while preserving the signal characteristics of high-frequency IMF components.

This phenomenon, combined with the analysis in Figure 10, shows that the EEMD-SE-IWTD denoised signal is overall closer to the clean signal, effectively retaining transient features and further enhancing the noise reduction effect.

## 5. Discussion

### 5.1. Complex Noise Source Analysis

The high-speed collision and penetration process between a high-speed moving object and an impacted object is a typical high-speed, transient, and complex dynamic event. During this process, the signal not only exhibits high-speed transient features but is also subjected to interference from complex noise caused by nonlinear and non-stationary factors. This complex noise significantly impacts the identification and analysis of the effective signal. Specifically, high-speed transient features are primarily concentrated within the 0–5 kHz frequency range, while complex noise manifests as resonant peaks and interference components beyond this range, further complicating signal processing.

Electromagnetic interference and mechanical vibrations are the primary sources of complex noise affecting accelerometers. Electromagnetic radiation and conducted interference can degrade the signal integrity, leading to false triggers or data loss. Mechanical vibration noise, caused by factors such as nonlinear spring damping characteristics and structural gaps, further compromises the signal accuracy and interferes with the accelerometer’s precise measurements. The presence of complex noise significantly hinders the identification and analysis of signal features. While existing noise reduction methods can eliminate some noise, they often fail to strike an optimal balance between retaining signal features and removing noise, particularly in the presence of significant complex noise, where traditional methods struggle to achieve satisfactory denoising results. Therefore, a new method is needed that ensures effective noise reduction while preserving essential signal features.

To address this issue, this paper proposes an EEMD-SE-IWTD combined noise reduction method, which aims to separate noise from signal features accurately, enhance denoising performance, and retain key transient information, thereby meeting the high-precision identification and analysis requirements of high-speed dynamic processes.

### 5.2. Indicator Comparison and Performance Analysis

Table 4 presents the performance metrics (average of 100 repeated tests) of different similar variants of noise reduction methods for noisy signals with an SNR*_in_* of white noise ranging from −10 dB to 10 dB, including the SNR*_out_*/dB, RMSE*_out_*, and CC*_out_* × 100%. A higher SNR*_out_*/dB, lower RMSE*_out_*, and CC*_out_* closer to 1 indicate better noise reduction performance. Specifically, the SNR*_out_* reflects the effectiveness of noise reduction, the RMSE*_out_* indicates the degree of waveform restoration, and the CC*_out_* measures the retention of waveform features.

From a vertical perspective, as the SNR*_in_* changes from −10 dB to 10 dB (with RMSE*_in_* values decreasing and CC*_in_* values increasing), the performance of all the methods shows significant improvement. This indicates that in signals with higher noise levels, the noise removal performance is poorer, while in signals with lower noise levels, the performance improves. Horizontally, EEMD-SE demonstrates relatively poor overall noise reduction performance. CWTD and IWTD exhibit excellent noise reduction performance in low SNR*_in_* noisy signals but perform poorly in high SNR*_in_* noisy signals, even performing worse than EEMD-SE in some cases. For EEMD-SE-CWTD, the noise reduction performance is poor at low SNR*_in_* but better at high SNR*_in_*. On the other hand, EEMD-SE-IWTD, except for the −10 dB SNR*_in_* case, where the noise reduction effect is worse than that of CWTD, performs very stably and with high accuracy in the rest of the cases. Especially at higher SNR*_in_* levels, where the SNR*_out_* reaches 21.13 dB, RMSE*_out_* drops to 570.83, and CC*_out_* reaches 0.9935, demonstrating its greater advantage in recovering signal accuracy and the correlation between signal and noise.

Table 5 demonstrates the performance metrics (averaged over 100 repetitions) of various advanced noise reduction methods for the SNR*_in_* ranging from −10 dB to 10 dB for white-noise-containing signals.

The performance of all the noise reduction methods improves as the SNR*_in_* increases. Compared to the CEEMDAN, VMD, and FSST methods, EEMD-SE-IWTD performs particularly well under different SNR*_in_* conditions, especially in low SNR*_in_* environments, demonstrating stronger noise reduction capability and higher signal recovery accuracy. Whether considering the three indices of SNR*_out_*, RMSE*_out_*, or CC*_out_*, the EEMD-SE-IWTD shows its superiority, proving its significant advantages in noise removal and signal recovery, making it an effective noise reduction method.

Table 4 and Table 5 show that the three indices, SNR*_out_*, RMSE*_out_*, and CC*_out_*, exhibit a synchronized trend. As the SNR*_out_* increases, the degree of waveform restoration and feature retention improves accordingly, reflected by the decrease in the RMSE*_out_* and an increase in the CC*_out_*. To visualize the superiority of the EEMD-SE-IWTD method, this paper uses the SNR*_out_* as the noise reduction performance index and plots error bar charts under different SNR*_in_* conditions, showing the SNR*_out_* for noise-containing signals with white noise, pink noise, and Laplace noise. The upper and lower bounds of the error bar charts represent the 95% confidence intervals of the mean values of the SNR*_out_*, further verifying the stability and superiority of the EEMD-SE-IWTD method under various noise conditions.

**Figure 14:**
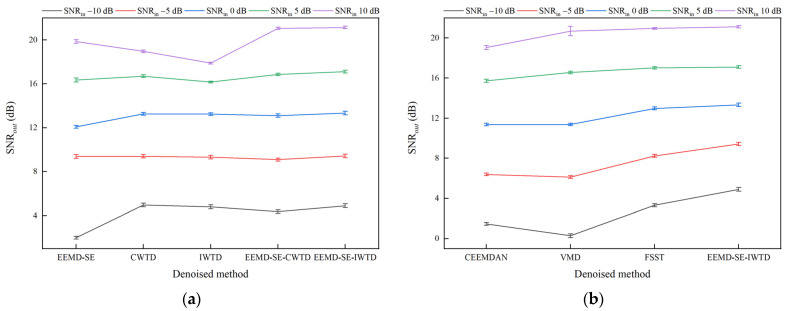
Noise reduction results for different SNR*_in_* white noise: (**a**) comparison of similar variants of noise reduction methods; and (**b**) comparison of advanced noise reduction methods.

**Figure 15:**
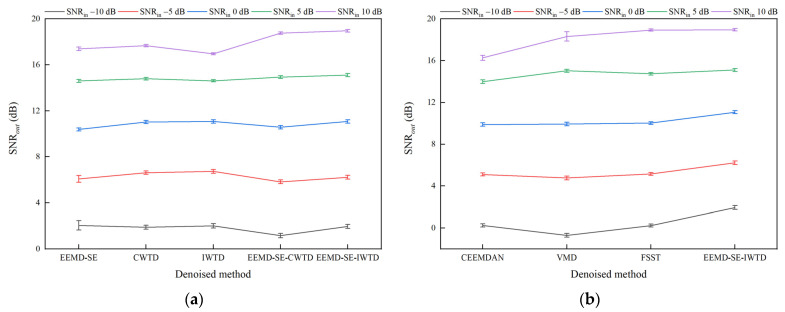
Noise reduction results for different SNR*_in_* pink noise: (**a**) comparison of similar variants of noise reduction methods; and (**b**) comparison of advanced noise reduction methods.

**Figure 16:**
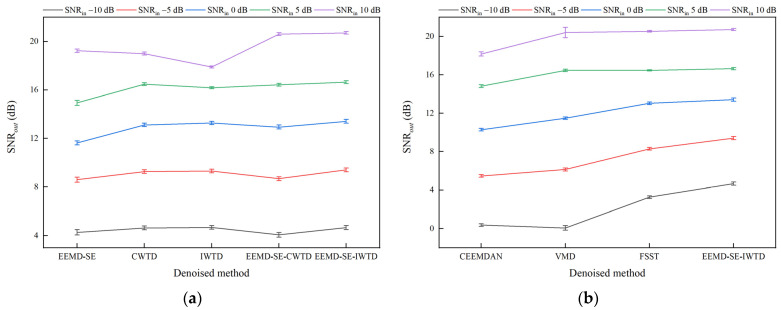
Noise reduction results for different SNR*_in_* Laplace noise: (**a**) comparison of similar variants of noise reduction methods; and (**b**) comparison of advanced noise reduction methods.

The results in Figure 14, Figure 15 and Figure 16 show that the EEMD-SE-IWTD method performs well under all types of noise conditions, especially for white noise and Laplace noise, demonstrating high noise reduction capability and strong stability. However, the EEMD-SE-IWTD method performs slightly worse than the IWTD method in pink noise conditions at −10 dB and −5 dB SNR*_in_*. The confidence intervals (95% upper and lower bounds) of the SNR*_out_* for the VMD method show large fluctuations across all noise conditions at 10 dB. The SNR*_out_* confidence intervals (95% upper and lower bounds) of the EEMD-SE method in powder noise conditions at −10 dB and −5 dB also show large fluctuations. In contrast, the other methods show smaller fluctuations. Overall, the EEMD-SE-IWTD performs the best in terms of noise reduction, with smaller fluctuations in its SNR*_out_* confidence intervals, demonstrating strong robustness. This suggests that the EEMD-SE-IWTD method is an effective approach that provides high-precision noise reduction while maintaining high stability.

This paper combines the EEMD, SE, and IWTD methods to achieve the best overall noise reduction performance. This is because each of the individual methods contributes to the overall result. Table 6 presents the analysis of the advantages of the method proposed in this paper.

### 5.3. Time Complexity Analysis

To further analyze the performance of the proposed method and the method used in Section 3.2.2, the algorithm’s efficiency was evaluated using a noise-containing signal with 0 dB white noise as the reference experimental data. The time complexity (TC) was determined, and the algorithm’s running time was recorded. Due to the varying running times in each experiment, each set of data was executed 100 times, and the average was calculated to obtain a more accurate result.

Ensemble empirical mode decomposition (EEMD), which involves performing M empirical mode decompositions (EMDs), has a time complexity that can be approximated as *O*(*MN* log *N*) [33]. The main computational aspect of sample entropy (SE) involves a double loop, where the outer loop traverses each embedding vector and the inner loop compares the distances with all the other vectors, resulting in an overall complexity of *O*(*N*^2^) [34]. In classical wavelet thresholding noise reduction, the main computational effort comes from fast wavelet decomposition and reconstruction, which has a linear complexity of *O*(*N*) [35]. The improved wavelet threshold noise reduction method only optimizes the threshold selection, so the complexity remains *O*(*N*). In summary, the overall complexity of both EEMD-SE-CWTD and EEMD-SE-IWTD can be expressed as *O*(*MN* log *N*) + *O*(*N*^2^) + *O*(*N*).

For other noise reduction methods, CEEMDAN adaptively superimposes white noise of a specific intensity *M* times during the decomposition of the residual signal, so its complexity is again *O*(*MN* log *N*). VMD has a complexity of approximately *O*(2*N* log_2_ (2*N*)) [36]. The synchrosqueezed transform, based on the short-time Fourier transform (STFT), adds instantaneous frequency estimation and coefficient redistribution to the STFT. The time complexity of STFT is *O*(*NK* log_2_ *K*), where *K* is the length of the window [37]. The instantaneous frequency estimation iterates through all the time–frequency points, resulting in a complexity of *O*(*KN*). At the same time, the synchrosqueezed process redistributes the instantaneous frequency values to the new time–frequency mesh, also with a complexity of *O*(*KN*). Therefore, the overall complexity of the synchrosqueezed transform based on the short-time Fourier transform can be expressed as *O*(*NK* log_2_ *K*) + *O*(*KN*). A comparison of the time complexity (TC) and running time (all algorithms are implemented using Python 3.8.10 (Python Software Foundation, Beaverton, OR, USA)) of various denoising methods presented in this paper is given in Table 7.

By combining the noise reduction results in Table 4 and Table 5 with the data in Table 7, it can be observed that although the EEMD-SE-IWTD method has a relatively long running time (0.829889 s), it provides the best overall noise reduction performance compared to the other seven methods. Methods such as VMD (0.188654 s) and CEEMDAN (0.425694 s) offer certain computational speed advantages, but they do not outperform EEMD-SE-IWTD in terms of noise removal effectiveness. The relationship between computational speed and noise reduction effectiveness shows that algorithms typically achieve excellent noise reduction at the cost of increased processing time. Therefore, although the EEMD-SE-IWTD method has a longer running time, its noise reduction performance is optimal, indicating that the method prioritizes enhancing noise reduction capability over computational speed in its application.

### 5.4. Generalization Test Analysis of Noise Reduction Methods

**Figure 17:**
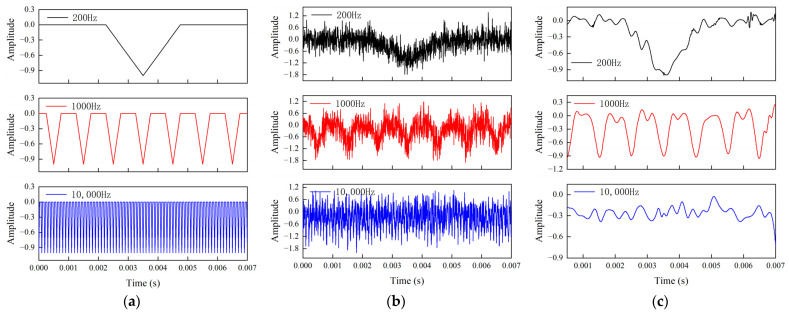
Triangle wave signal display: (**a**) clean triangular wave signal; (**b**) noise-containing signal; and (**c**) denoised result.

**Figure 18:**
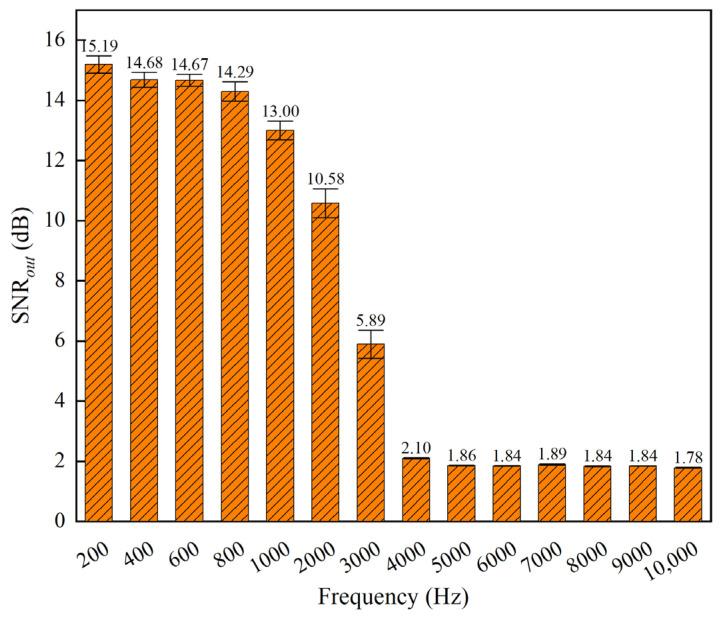
Comparison of SNR*_out_* for signals with different main frequencies.

In order to deeply investigate the performance boundaries of the method presented in this paper, test experiments were designed to analyze the correlation between the main frequency of the signal and the noise reduction effect. To verify the sensitivity of the method to changes in the main frequency of the signal, signals with main frequencies of 200 Hz, 400 Hz, 600 Hz, 800 Hz, 1000 Hz, 2000 Hz, 3000 Hz, 4000 Hz, 5000 Hz, 6000 Hz, 7000 Hz, 8000 Hz, 9000 Hz, and 10,000 Hz were generated. These were triangle wave analog signals with a sampling rate of 200 kHz, where the pulse width of each triangle wave was half its period length (e.g., for a 1000 Hz signal with a period of 1 ms, the pulse width was 0.5 ms). The crest amplitude was set to 1, and the signal length was 7 ms (1400 samples in total), ensuring consistency with the shock signal conditions used in the multiple penetration experiments described in Section 3.2.1. These signals were then superimposed with 0 dB SNR*_in_* white noise to create noise-containing signals for the test set. Figure 17 illustrates a typical multi-layer triangular wave signal, the noise-containing signal, and its noise reduction results. Figure 18 compares the noise reduction performance of signals with different main frequencies.

The experimental results show that the method presented in this paper achieves excellent noise reduction for signals with main frequencies below 1000 Hz. However, as the main frequency increases from 1000 Hz to 4000 Hz, the noise reduction effect significantly attenuates. The performance is poorest for high-frequency signals above 5000 Hz, where the SNR*_out_* is reduced to less than 1.9 dB. This indicates a substantial decrease in the noise reduction capability as the main frequency increases.

To investigate the reasons for the attenuation of the noise reduction performance, experiments are designed to explore the correlation between the sample length, the number of effective signal cycles, and the noise reduction effectiveness.

**Figure 19:**
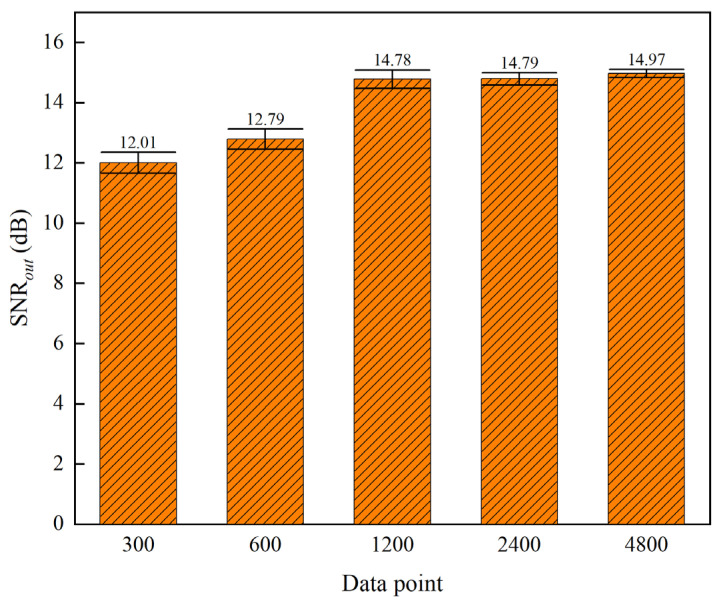
Comparison of SNR*_out_* for signals with different sample lengths.

First, an experiment is conducted to examine the correlation between sample length and noise reduction performance. With the number of effective signal cycles held constant, the sample length of the signal is adjusted, and the results are analyzed with the number of sampling points as the horizontal axis. In the experiments, clean signals with sampling lengths of 300, 600, 1200, 2400, and 4800 are generated. Each signal contains a triangular wave with three cycles, a pulse width of 50% of the cycle length, and a peak amplitude of 1. The 0 dB SNR*_in_* with white noise is then superimposed on these signals. The noise reduction results for signals with different sample lengths are shown in Figure 19.

The experimental results shown in Figure 19 demonstrate that the method presented in this paper still achieves better noise reduction performance when the horizontal axis represents the number of sampling points. This indicates that the degradation of the noise reduction performance is not caused by variations in the dominant frequency. Further experiments reveal that, under the condition of a constant number of effective signal cycles, a longer sampling length results in better noise reduction, and the confidence interval of the output signal-to-noise ratio (SNR*_out_*) gradually narrows. As shown in Figure 19, when the number of sampling points is 300, the SNR*_out_* is 12.01 dB, whereas when the number of sampling points is increased to 4800, the SNR*_out_* improves to 14.97 dB, showing a significant improvement.

**Figure 20:**
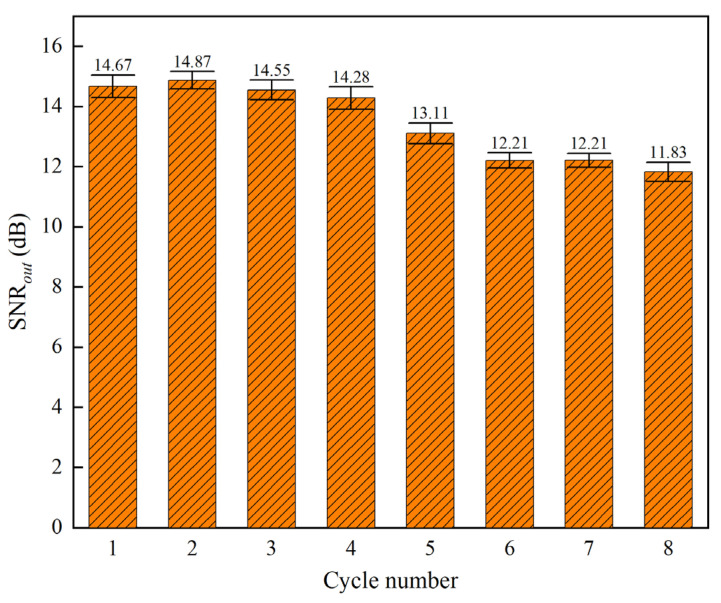
Comparison of SNR*_out_* for signals with different numbers of triangular wave periods at a sample length of 1000.

Next, an experiment was conducted to examine the correlation between the number of effective signal cycles and the noise reduction performance. The experiment tested the effect of different numbers of effective signal cycles on the noise reduction under a fixed sampling length. In this experiment, the sampling length was fixed at 1000, and triangular wave signals containing one to eight cycles were generated. 0 dB SNR*_in_* with white noise was then superimposed on these signals. After noise reduction, the results are shown in Figure 20. The experimental results indicate that, with 1000 sampling points, triangular wave signals containing four cycles or fewer can achieve the ideal noise reduction effect. That is, the best noise reduction performance of the method presented in this paper occurs when the length of a single cycle of the effective signal is no less than 250 sample points.

From the experimental results above, it can be observed that the sample entropy values of all the IMF components after EEMD increase as the number of valid signal cycles increases under a fixed sampling length. In particular, the entropy values of IMF components containing valid features increase and tend to approach the noise level, which raises the probability of misclassification when the sample entropy threshold (SE) is applied. Therefore, as shown in Figure 18, under the condition of a fixed sampling length, the higher the main frequency of the signal, the worse the noise reduction effect.

In summary, the upper limit of the noise reduction performance of the method presented in this paper (SE = 0.30) occurs when the length of a single cycle of the effective signal to be processed is no less than 250 sampling points. For acceleration signals with high-speed transient complex features, this method is effectively applicable to the noise reduction of such signals, as these signals have fewer signal cycles and more sampling points. Under other conditions, if the characteristics of the desired noise-reduced signal change, the SE value can be reset through the SE value mechanism and sensitivity analysis described in Section 4.2 to further optimize the noise reduction effect.

### 5.5. Verification of Noise Reduction on Real Acceleration Signals

**Figure 21:**
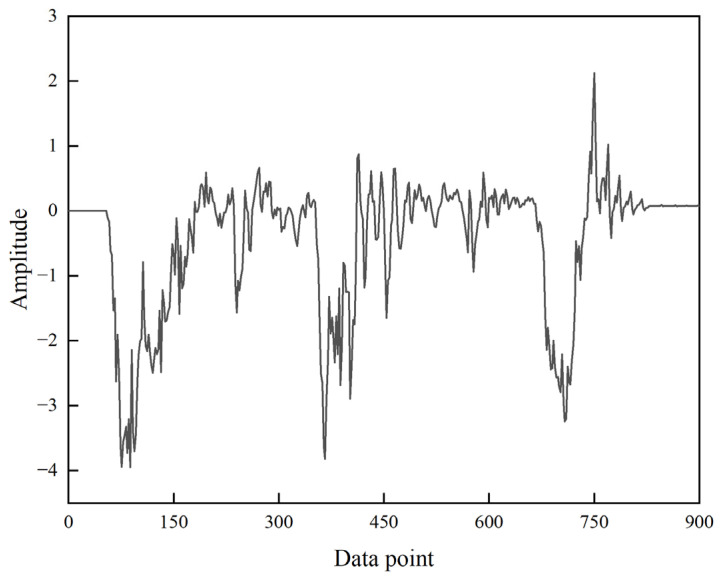
Measured acceleration signal curve.

To verify the effectiveness and robustness of the EEMD-SE-IWTD method proposed in this paper in real-world engineering scenarios, a set of measured high-speed transient complex penetration acceleration signals from a high-speed moving object penetrating through a three-layer concrete target is introduced. The measurements are conducted using a high-g accelerometer. Figure 21 shows the waveforms of the original acceleration signals (which have been Z-score normalized), with a length of 900 sampling points. It can be observed that the measured signal contains more complex background noise and interference compared to the impact data from the experimental apparatus (Figure 9a clean multiple impact signals).

**Figure 22:**
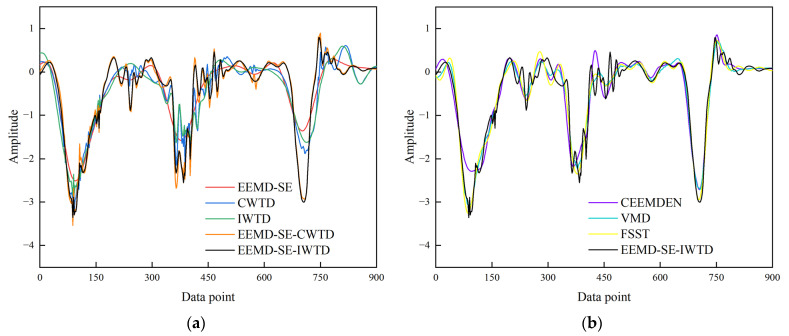
Noise reduction results for different methods: (**a**) comparison of similar variants of noise reduction methods; and (**b**) comparison of advanced noise reduction methods.

The method proposed in this paper, along with similar variants of noise reduction methods and advanced noise reduction methods, is applied to the measured signal. The noise reduction results are shown in Figure 22.

Figure 22a shows the noise reduction results of the method proposed in this paper, along with similar variants of noise reduction methods. The EEMD-SE method reduces noise excessively, leading to signal distortion. The CWTD and IWTD methods suppress some noise but cause significant amplitude attenuation of features, particularly in the third layer of the waveform. The EEMD-SE-CWTD method improves the result but still exhibits large burr oscillation noise, making it less effective than the method presented in this paper, which strikes a better balance between feature preservation and noise smoothing. Figure 22b presents the noise reduction results of this paper’s method and the advanced noise reduction methods. The CEEMDAN method is overly smooth, reducing the feature amplitude and causing signal distortion, while the VMD and FSST methods provide better noise reduction, though some smoothing is still evident in the details.

**Figure 23:**
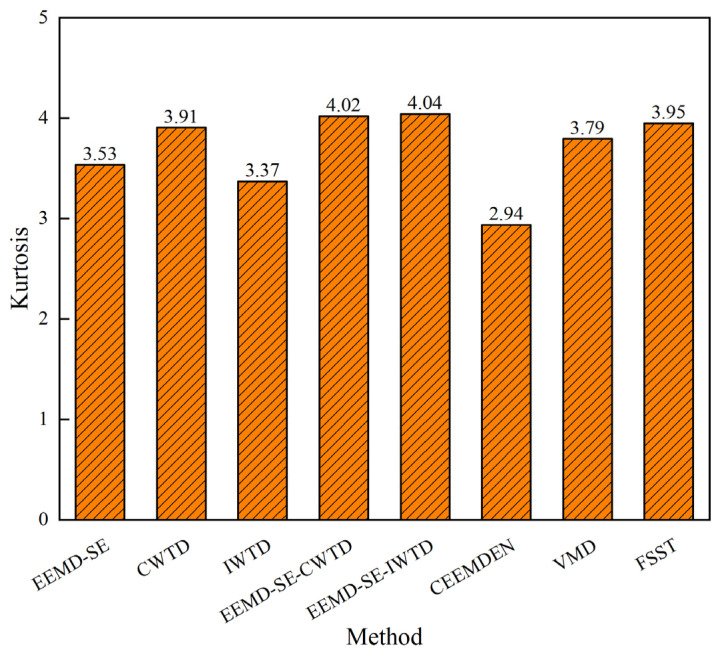
Comparison of kurtosis for signals with different noise reduction methods.

Since the baseline clean signal of the measured data is unknown, the SNR*_out_*, RMSE*_out_*, CC*_out_*, and other indicators cannot be calculated. Therefore, kurtosis is used as an evaluation index to measure the regularity of the signal waveform after noise reduction. In the analysis of high-speed through-acceleration signals, higher kurtosis values typically indicate that the signals contain more significant transient shock features, which are crucial for identifying the number of penetration layers. As shown in Figure 23, the processed signal exhibits the highest kurtosis, demonstrating that the EEMD-SE-IWTD method effectively preserves the transient features generated by the penetration of the high-speed moving body into the impacted target while suppressing the noise. This confirms the effectiveness and robustness of the method.

Through comparison, the noise reduction effect of the method (EEMD-SE-IWTD) proposed in this paper is the most effective, with its noise-reduced signal fully and clearly preserving the overload waveforms generated by the high-speed moving body as it penetrates each layer of the impacted target. The high-fidelity waveforms after noise reduction, based on this method, provide a high-quality dataset for the subsequent target characterization, with applications primarily in the following two areas.

In penetration event identification and layer discrimination, based on the clear signal waveform characteristics after noise reduction (amplitude, pulse width, and pulse interval), the amplitude threshold and pulse counting algorithms can be set to automatically and accurately determine the number of penetration layers. Additionally, the ability to accurately identify the number of layers can be directly integrated into the control logic to ensure that follow-up actions are triggered at the optimal time after penetrating a specific number of layers, thus enhancing the intelligence and reliability of the control system [38].

In terms of dynamic inversion and parameter extraction of the penetration process, high-fidelity waveform characteristics provide the possibility of inverting dynamic parameters of the penetration process. For example, the pulse width can be related to the interaction time between two objects, the amplitude can reflect the impact overload strength, and the pulse time interval can correspond to the time needed to penetrate neighboring impacted targets. Based on these physical quantities, key parameters such as the penetration depth, velocity decay curve, average velocity, and others can be accurately recovered, providing data support for analyzing the dynamic response characteristics of high-speed moving bodies [39].

### 5.6. Advantages

The EEMD-SE-IWTD combined noise reduction method proposed in this paper demonstrates significant advantages in processing multi-layer target plate penetration acceleration signals, particularly in balancing noise removal and signal feature retention.

For high-speed transient complex feature multi-layer acceleration signals, an EEMD-SE-IWTD combined noise reduction method is developed. Specifically, by applying a sample entropy threshold (SE = 0.3), the complex noise-dominated components and effective transient feature components are precisely separated, thereby enhancing the objectivity and accuracy of modal segmentation. Additionally, an improved wavelet threshold denoising method is applied to the complex noise-dominated components, effectively eliminating high-frequency interference while preserving the effective impact features.

The EEMD-SE-IWTD method performs well under various simulated noise conditions, especially in white noise and Laplace noise, demonstrating high noise reduction capability and strong stability. However, for pink noise with low SNR*_in_* (−10, −5 dB), the noise reduction effect is weaker compared to IWTD. Overall, the noise reduction performance, waveform fidelity, and feature retention of the proposed method significantly outperform those of the comparison methods. Compared to other noise reduction methods, the EEMD-SE-IWTD method effectively removes high-frequency noise and interference while preserving signal features, ensuring the stability and reliability of the noise reduction process.

The EEMD-SE-IWTD method shows significant advantages in noise reduction for real engineering scenarios. By reducing noise in measured high-speed transient penetration acceleration signals, this method effectively suppresses noise while preserving the transient features generated by a high-speed moving body penetrating the target. Compared to similar variants and state-of-the-art noise reduction methods, EEMD-SE-IWTD achieves a better balance between feature preservation and noise smoothing, avoiding signal distortion and feature amplitude attenuation caused by excessive smoothing. The results show that the EEMD-SE-IWTD method yields the highest kurtosis value for the processed signal, indicating its effectiveness in preserving significant transient shock features. These features are crucial for identifying penetration layers and extracting dynamic parameters. Based on the noise-canceled high-fidelity waveforms, this method provides high-quality data for subsequent target characterization, layer discrimination, and dynamic parameter inversion, demonstrating its strong advantages in practical applications.

In summary, the EEMD-SE-IWTD combined noise reduction method demonstrates superior performance by precisely separating noise from effective signal features, significantly improving noise reduction, maintaining signal features, and employing a reasonable evaluation method. This method not only offers theoretical advantages but also shows substantial performance improvements in applications with broad potential for future use.

### 5.7. Further Research Directions

Although the EEMD-SE-IWTD combined noise reduction method proposed in this paper has achieved good results in processing multi-layer through acceleration signals, some limitations remain. Future research can further explore and optimize the following aspects.

First, the scene dependency of sample entropy. The current sample entropy threshold (SE = 0.3) is set based on the typical signal structure characteristics (sample length, number of active waveform cycles) of multi-layer acceleration signals. In this scene, this threshold effectively separates noise from valid signals. However, when applied to other signal structures, the applicability of the threshold may be limited. Since the structural characteristics of different signals vary significantly, it is necessary to recalibrate the sample entropy threshold for different scenarios to ensure it adapts to the characteristics of the target signal, thereby enhancing the adaptability and universality of the noise reduction effect. Future research could explore more flexible methods for selecting the sample entropy threshold or combine adaptive algorithms to dynamically adjust the threshold based on the signal’s frequency characteristics.

Second, the computational complexity is relatively high. The EEMD-SE-IWTD method demonstrates significantly superior noise reduction performance compared to other approaches, particularly in complex noise environments. However, it exhibits high computational complexity due to multiple steps, including signal decomposition, sample entropy calculation, and wavelet threshold denoising. This results in considerable time complexity, limiting its performance in real-time applications. In the future, the computational efficiency can be improved by optimizing the algorithms and integrating hardware acceleration technology.

By thoroughly studying these limitations and gradually overcoming them, the method described in this paper is expected to play a greater role in a wider range of applications, particularly in high-precision signal processing and practical applications in complex noise environments.

## 6. Conclusions

- For multi-layer through-acceleration signals with high-speed transient complex features, this paper proposes an EEMD-SE-IWTD combined noise reduction method. This method uses EEMD to decompose the signal and obtain multi-layer IMF components. The IMF components are then divided using a sample entropy threshold (SE = 0.3), with high-entropy IMF components processed using IWTD to retain effective impact features. IMF components in the low-entropy region are not processed and are retained as effective transient feature components, ultimately reconstructing the denoised signal.
- This paper also proposes a method for simulating high-speed transient complex feature multi-layer through-acceleration signals. Impact experiments are conducted using existing equipment to obtain clean multi-layers in acceleration signals, which are then overlaid with Gaussian white noise, pink noise, and Laplace noise sequences at different SNR*_in_* values (−10 dB, −5 dB, 0 dB, 5 dB, 10 dB) to generate simulated signals with varying SNR*_in_*.
- To validate the effectiveness of the proposed method, noise reduction processing was performed on simulated complex multi-layer through-acceleration signals with different Gaussian white noise, pink noise, and Laplace noise sequences. The noise reduction performance of the EEMD-SE-IWTD method was compared with that of similar variants (EEMD-SE, CWTD, IWTD, and EEMD-SE-CWTD) and advanced noise reduction methods (CEEMDAN, VMD, and FSST). The experimental results show that the proposed method exhibits excellent noise reduction performance under signals containing white noise and Laplace noise. However, its noise reduction effectiveness is inferior to IWTD under signals containing pink noise at SNR*_in_* levels of −10 dB and −5 dB. Overall, the proposed method effectively preserves transient features while enhancing noise reduction performance, enabling efficient noise reduction for noisy signals. The main improvements are as follows. After EEMD decomposition, high-frequency spikes still exist in the high-entropy-region IMF components after CWTD denoising. At the same time, IWTD effectively eliminates these high-frequency spikes.
- This paper addresses the issues in existing EEMD methods, where complex noise dominates the IMF decomposition, the division of effective feature components is overly subjective, and the complete removal of high-frequency components leads to unclear features. Through experimental verification of noise reduction in multi-layer penetration acceleration signals, the proposed method demonstrates higher kurtosis than other approaches. It preserves more effective transient features while achieving superior noise reduction performance. Applying this method to real-world signals enables both penetration layer identification and reconstruction analysis of the penetration process, providing significant practical value.

Future research can further enhance the applicability of this method by developing an adaptive modal threshold division mechanism. Additionally, improving the algorithm to enhance the computational efficiency could make it suitable for real-time detection. The method proposed in this paper effectively addresses the issue of high-frequency resonant noise masking effective transient features in acceleration signals. This method can also be applied to fields characterized by significant noise signals, such as mechanical fault diagnosis and geological exploration. It will be instrumental in the development of more dependable sensor systems suitable for complex dynamic environments.

## Figures and Tables

**Table 1:** Comparison of similar variants of noise reduction methods.

No.	Method	Parameters
1	EEMD-SE	IMF discarding with entropy > 0.3
2	CWTD	db8; general threshold principle; soft threshold
3	IWTD	db8; improved threshold principle; soft threshold
4	EEMD-SE-CWTD	IMF wavelet threshold denoising with entropy > 0.3; db8; general threshold principle; soft threshold
5	EEMD-SE-IWTD	IMF wavelet threshold denoising with entropy > 0.3; db8; improved threshold principle; soft threshold

**Table 2:** IMF component SE values, center frequencies, physical significance, and processing methods.

IMF	SE	Center Frequency	Physical Significance	Handling Method
IMF1	1.5322	72.234 kHz	Sensor high-frequency resonance noise	IWTD
IMF2	1.0100	19.985 kHz	Sensor high-frequency resonance noise	IWTD
IMF3	0.6213	10.710 kHz	Bullet target dynamic response oscillation	IWTD
IMF4	0.4101	5.139 kHz	Bullet target dynamic response oscillation	IWTD
IMF5	0.0998	1.570 kHz	The main component of impact acceleration	Reserve
IMF6	0.0434	0.571 kHz	The main component of impact acceleration	Reserve
IMF7	0.0237	0.285 kHz	The main component of impact acceleration	Reserve

**Table 3:** The denoising metrics for different SE values.

SE	Output	SNR*_in_*				
		−10 dB	−5 dB	0 dB	5 dB	10 dB
SE = 0.10	SNR*_out_*/dB	4.82	9.39	13.38	16.39	18.09
	RMSE*_out_*	3749.53	2210.86	1393.64	985.36	809.64
	CC*_out_* × 100%	80.00%	91.39%	96.26%	98.08%	98.69%
SE = 0.15	SNR*_out_*/dB	4.87	9.42	13.48	16.38	18.67
	RMSE*_out_*	3718.54	2202.05	1377.63	986.15	763.07
	CC*_out_* × 100%	79.98%	91.39%	96.35%	98.07%	98.82%
SE = 0.20	SNR*_out_*/dB	4.76	9.46	13.44	16.40	20.56
	RMSE*_out_*	3777.32	2192.56	1385.10	983.05	614.11
	CC*_out_* × 100%	78.65%	91.34%	96.28%	98.08%	99.24%
SE = 0.25	SNR*_out_*/dB	4.77	9.40	13.34	16.67	20.96
	RMSE*_out_*	3767.66	2210.86	1401.57	954.58	582.26
	CC*_out_* × 100%	79.40%	91.13%	96.23%	98.21%	99.33%
SE = 0.30	SNR*_out_*/dB	4.90	9.42	13.34	17.11	21.13
	RMSE*_out_*	3710.01	2201.76	1403.41	907.78	570.83
	CC*_out_* × 100%	79.89%	91.38%	96.21%	98.38%	99.35%
SE = 0.35	SNR*_out_*/dB	4.56	9.27	12.73	16.97	20.94
	RMSE*_out_*	3873.53	2247.72	1504.17	921.88	583.63
	CC*_out_* × 100%	78.89%	91.19%	95.77%	98.34%	99.32%
SE = 0.40	SNR*_out_*/dB	3.38	8.06	12.38	17.07	21.02
	RMSE*_out_*	4449.62	2594.14	1565.72	911.94	577.84
	CC*_out_* × 100%	74.79%	88.50%	95.45%	98.38%	99.34%
SE = 0.45	SNR*_out_*/dB	2.43	7.40	12.26	16.90	20.97
	RMSE*_out_*	4922.41	2777.89	1586.02	929.46	581.43
	CC*_out_* × 100%	71.40%	87.48%	95.32%	98.32%	99.33%
SE = 0.50	SNR*_out_*/dB	2.42	7.33	12.34	17.02	20.99
	RMSE*_out_*	4925.96	2800.10	1571.89	917.83	580.87
	CC*_out_* × 100%	71.65%	87.14%	95.40%	98.36%	99.33%

**Table 4:** Performance metrics of various similar variants of noise reduction methods under different SNR*_in_* with white noise conditions.

Input	Method	SNR*_out_*/dB	RMSE*_out_*	CC*_out_* × 100%
SNR*_in_*/dB: −10 RMSE*_in_*: 20,838.55 CC*_in_* × 100%: 21.13%	EEMD-SE	2.00	5167.41	69.32%
	CWTD	4.97	3678.59	80.73%
	IWTD	4.80	3752.32	79.03%
	EEMD-SE-CWTD	4.36	3945.77	77.63%
	EEMD-SE-IWTD	4.90	3710.01	79.89%
SNR*_in_*/dB: −5 RMSE*_in_*: 11,472.54 CC*_in_* × 100%: 38.14%	EEMD-SE	9.37	2216.03	90.58%
	CWTD	9.40	2209.67	91.16%
	IWTD	9.32	2229.09	91.21%
	EEMD-SE-CWTD	9.09	2286.21	90.73%
	EEMD-SE-IWTD	9.42	2201.76	91.38%
SNR*_in_*/dB: 0 RMSE*_in_*: 6335.16 CC*_in_* × 100%: 61.41%	EEMD-SE	12.08	1619.19	94.85%
	CWTD	13.27	1412.15	96.14%
	IWTD	13.25	1415.61	96.14%
	EEMD-SE-CWTD	13.11	1440.31	96.06%
	EEMD-SE-IWTD	13.34	1403.41	96.21%
SNR*_in_*/dB: 5 RMSE*_in_*: 3665.36 CC*_in_* × 100%: 80.51%	EEMD-SE	16.34	994.88	98.03%
	CWTD	16.70	951.58	98.21%
	IWTD	16.16	1011.36	97.96%
	EEMD-SE-CWTD	16.85	934.88	98.30%
	EEMD-SE-IWTD	17.11	907.78	98.38%
SNR*_in_*/dB: 10 RMSE*_in_*: 2051.30 CC*_in_* × 100%: 92.51%	EEMD-SE	19.85	662.70	99.12%
	CWTD	18.96	733.48	98.92%
	IWTD	17.89	828.69	98.62%
	EEMD-SE-CWTD	21.06	575.22	99.35%
	EEMD-SE-IWTD	21.13	570.83	99.35%

**Table 5:** Performance metrics of various advanced noise reduction methods under different SNR*_in_* with white noise conditions.

Input	Method	SNR*_out_*/dB	RMSE*_out_*	CC*_out_* × 100%
SNR*_in_*/dB: −10 RMSE*_in_*: 20,838.55 CC*_in_* × 100%: 21.13%	CEEMDAN	1.46	5501.23	66.10%
	VMD	0.28	6319.40	61.69%
	FSST	3.32	4445.76	74.40%
	EEMD-SE-IWTD	4.90	3710.01	79.89%
SNR*_in_*/dB: −5 RMSE*_in_*: 11,472.54 CC*_in_* × 100%: 38.14%	CEEMDAN	6.38	3122.72	85.11%
	VMD	6.11	3222.13	84.14%
	FSST	8.23	2524.39	89.39%
	EEMD-SE-IWTD	9.42	2201.76	91.38%
SNR*_in_*/dB: 0 RMSE*_in_*: 6335.16 CC*_in_* × 100%: 61.41%	CEEMDAN	11.36	1759.90	94.39%
	VMD	11.38	1755.71	94.36%
	FSST	12.98	1462.24	96.06%
	EEMD-SE-IWTD	13.34	1403.41	96.21%
SNR*_in_*/dB: 5 RMSE*_in_*: 3665.36 CC*_in_* × 100%: 80.51%	CEEMDAN	15.72	1066.74	97.77%
	VMD	16.56	967.32	98.19%
	FSST	17.01	917.56	98.37%
	EEMD-SE-IWTD	17.11	907.78	98.38%
SNR*_in_*/dB: 10 RMSE*_in_*: 2051.30 CC*_in_* × 100%: 92.51%	CEEMDAN	19.05	728.72	98.94%
	VMD	20.69	627.36	99.11%
	FSST	20.96	581.52	99.33%
	EEMD-SE-IWTD	21.13	570.83	99.35%

**Table 6:** EEMD-SE-IWTD combined noise reduction mechanism.

Individual Method	Key Contributions	Combined Benefits
EEMD	Adaptively generates physically meaningful IMF components, suppressing end-point effects and modal aliasing.	Provides high-precision time–frequency decomposition of IMF for noise recognition.
SE	IMF complexity classification based on sample entropy: the high-entropy zone corresponds to dominant complex feature noise, and the low-entropy zone corresponds to dominant effective transient features.	Accurately separates complex noise-dominated and effective transient feature components.
IWTD	Improves the wavelet threshold principle, reduces noise in high-frequency IMF components, and retains effective features.	Eliminates high-frequency glitches caused by traditional threshold principles.
Combined mechanism	EEMD decomposes the signal, SE classifies the IMF components, and IWTD processes the noise.	Retains transient features and improves overall noise reduction performance.

**Table 7:** Comparison of TC and running time.

Method	TC	Running Time
EEMD-SE	*O*(*MN* log *N*) + *O* (*N*^2^)	0.787744 s
CWTD	*O* (*N*)	0.003093 s
IWTD	*O* (*N*)	0.003217 s
EEMD-SE-CWTD	*O* (*MN* log *N*) + *O* (*N*^2^) + *O* (*N*)	0.802135 s
CEEMDAN	*O* (*MN* log *N*)	0.425694 s
VMD	*O* (2*N* log_2_ (2*N*))	0.188654 s
FSST	*O* (*NK* log_2_ *K*) + *O*(*KN*)	0.074771 s
EEMD-SE-IWTD	*O* (*MN* log *N*) + *O* (*N*^2^) + *O* (*N*)	0.829889 s

## Data Availability

All data generated or analyzed during this study are included in the article. The data and intellectual property rights belong to Nanjing University of Science and Technology. The original contributions presented in this study are included in the article. Further inquiries regarding the data and intellectual property can be directed to the corresponding author(s).
